# Supplementary material for: Integrative network analysis of early-stage lung adenocarcinoma identifies aurora kinase inhibition as interceptor of invasion and progression
Source: Nat Commun. 2022 Mar 24;13:1592. doi: 10.1038/s41467-022-29230-7 (PMC8948234; doi:10.1038/s41467-022-29230-7)
Supplement: Supplementary file 1 — Supplementary Information [file 41467_2022_29230_MOESM1_ESM.pdf]

Supplementary Information for

Integrative Network Analysis of Early-Stage Lung Adenocarcinoma Identifies Aurora Kinases Inhibition as Interceptor of Invasion and Progression

Seungyeul Yoo[1,2,3,#], Abhilasha Sinha[4,5,#], Dawei Yang[4,5,6], Nasser K. Altorki[7], Radhika Tandon[8], Wenhui Wang[1,2], Deebly Chavez[4], Eunjee Lee[1,2,3], Ayushi S. Patel[4,5,9], Takashi Sato[4,5,10,11], Ranran Kong[4,5,12], Bisen Ding[4,13], Eric E. Schadt[1,2,3,5], Hideo Watanabe[1,2,4,5], Pierre P. Massion[14,^], Alain C. Borczuk[15], Jun Zhu[1,2,3,5,*], and Charles A. Powell[4,5,*]

# These authors contributed equally

***These authors jointly supervised this work**

^ Deceased

Contacts: jun.zhu@mssm.edu, charles.powell@mssm.edu

1. Department of Genetics and Genomic Sciences, Icahn School of Medicine at Mount Sinai, New York, NY, USA
2. Icahn Institute for Data Science and Genomic Technology, New York, NY, USA
3. Sema4, Stamford, CT, USA
4. Division of Pulmonary, Critical Care and Sleep Medicine, Icahn School of Medicine at Mount Sinai, New York, NY, USA
5. Tisch Cancer Institute, Icahn School of Medicine at Mount Sinai, New York, NY, USA
6. Department of Pulmonary and Critical Care Medicine, Zhongshan Hospital, Fudan University, Shanghai, China.
7. Department of Cardiothoracic Surgery, Weill Cornell Medicine-New York Presbyterian Hospital, New York, NY, USA
8. School of Medicine, St. George's University, West Indies, Grenada
9. Vileck Institute of Graduate Biomedical Sciences, New York University School of Medicine, New York, NY, USA
10. Division of Pulmonary Medicine, Department of Medicine, Keio University School of Medicine, Tokyo, Japan
11. Department of Respiratory Medicine, Kitasato University School of Medicine, Sagamihara, Japan
12. Department of Thoracic Surgery, The Second Affiliated Hospital of Medical School, Xi'an Jiaotong University, Xi'an, Shaanxi, China
13. Key Laboratory of Birth Defects And Related Diseases of Women And Children of MOE, State Key Laboratory of Biotherapy, West China Second University Hospital, Sichuan University, Chengdu, Sichuan, China.
14. Department of Medicine, Vanderbilt University Medical Center, Nashville, TN, USA
15. Department of Pathology, Weill Cornell Medicine, New York, NY, USA

Supplementary Figures

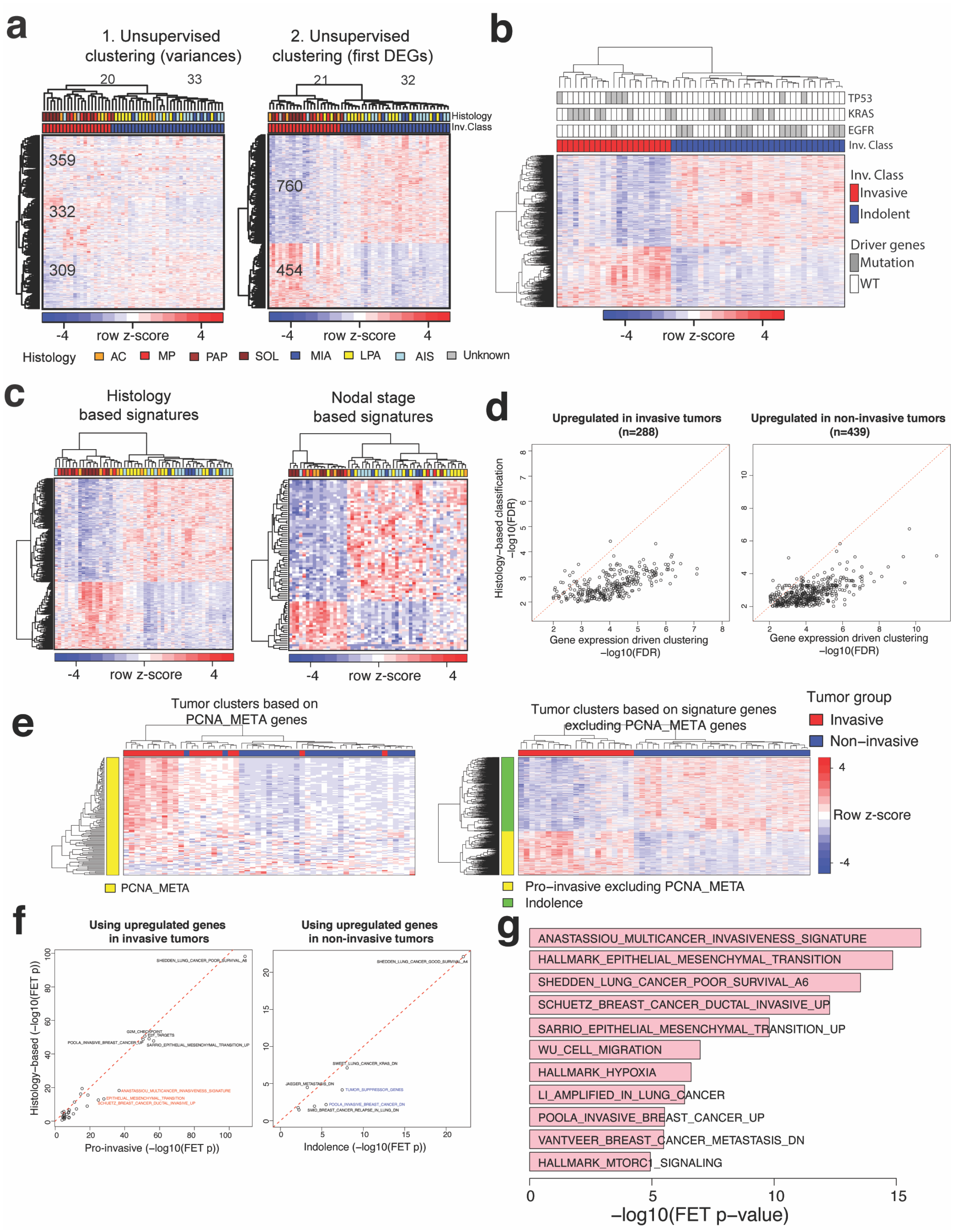

Supplementary Fig. 1. Identification of invasive signature genes.

a. Unsupervised clustering of 53 early-stage lung adenocarcinoma using top 1000 most varying genes (left) and another unsupervised clustering of the tumors using the DEGs between two groups separated by the most varying genes (right). Source data are provided in Source Data files.

b. Mutational landscape of 3 lung cancer driver genes (EGFR, KRAS, and TP53) among 53 early-stage lung adenocarcinoma. Source data are provided in Source Data files.

c. Differentially expressed genes based on histology (left) or nodal stage (right) of 53 early-stage lung adenocarcinoma. DEGs were determined using the same cutoffs (FC>1.5 & FDR<0.01). There were 313 genes up-regulated in tumors with aggressive histology (AC, MP, PAP, and SOL) compared to AIS/MIA/LPA tumors and 480 genes down-regulated in aggressive tumors. For node stage based DEGs, twenty-three genes were up-regulated and 57 genes were down-regulated in node positive tumors compared to no nodal metastasis tumors. Color code for the histology of each patient is same as in panel a. Source data are provided in Source Data files.

d. Gene expression differences of 727 common genes identified both from gene expression driven unsupervised clustering and histology-based clustering. Left: 288 common genes from pro-invasive and upregulated in histologically invasive tumors. Right: 439 common genes from indolence signature genes and upregulated genes in histologically non-invasive tumors. T-test FDRs of these genes between invasive and non-invasive tumors from the two approaches were compared. The detail statistics included in Supplementary Data 1.

e. Tumor clusters determined by meta-PCNA genes (left) and our signature genes excluding meta-PCNA genes. Source data are provided in Source Data files.

f. Functional enrichment analysis in MSigDB gene sets using signatures identified by unsupervised clustering using gene expression profiles and histology-based classification. Left: Genes upregulated in invasive tumors. Right: Genes upregulated in non-invasive

tumors. Fisher's exact test FDRs against the gene sets were compared between pro-invasive/indolence signature genes and histology based DEGs. Source data are provided in Source Data files.

g. Enriched gene sets within the unique in pro-invasive signature genes but not in histology based DEGs. One-sided FET p-values without multiple correction were log10 transformed. Source data are provided in Source Data files.

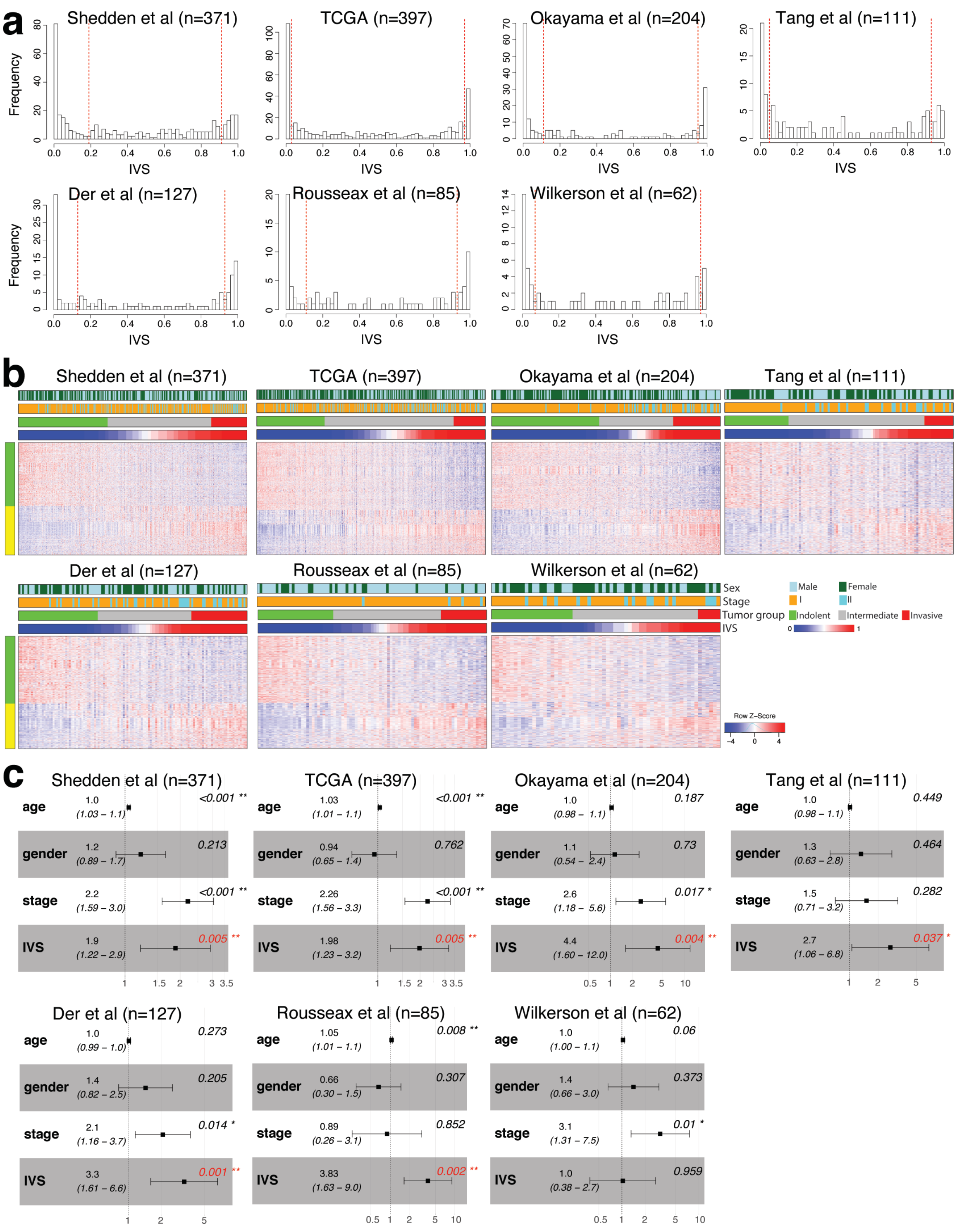

Supplementary Fig. 2. Invasiveness Score (IVS) associated with patient's survival

a. Distribution of IVS in 7 independent lung cancer cohorts (stage I and II only). Tumors were further clustered into high, middle, and low IVS groups based on local minima of IVS based on the histogram (bin size=0.025). Source data are provided in Source Data files.
b. Heatmap of expression of the signature genes of tumors in 7 cohorts. Samples were sorted from lowest IVS (left) to highest IVS (right).
c. Forest-plots showing significant association of IVS with patients' survival in multiple lung cancer cohorts. Hazard ratios with 95% confidence intervals IVS were measured with age, sex, and stage as covariates. Center dots indicate Hazard ratios and error bars indicate upper and lower 95% confidence intervals. Significant associations (unadjusted two-sided test p <0.05) are shown in red.

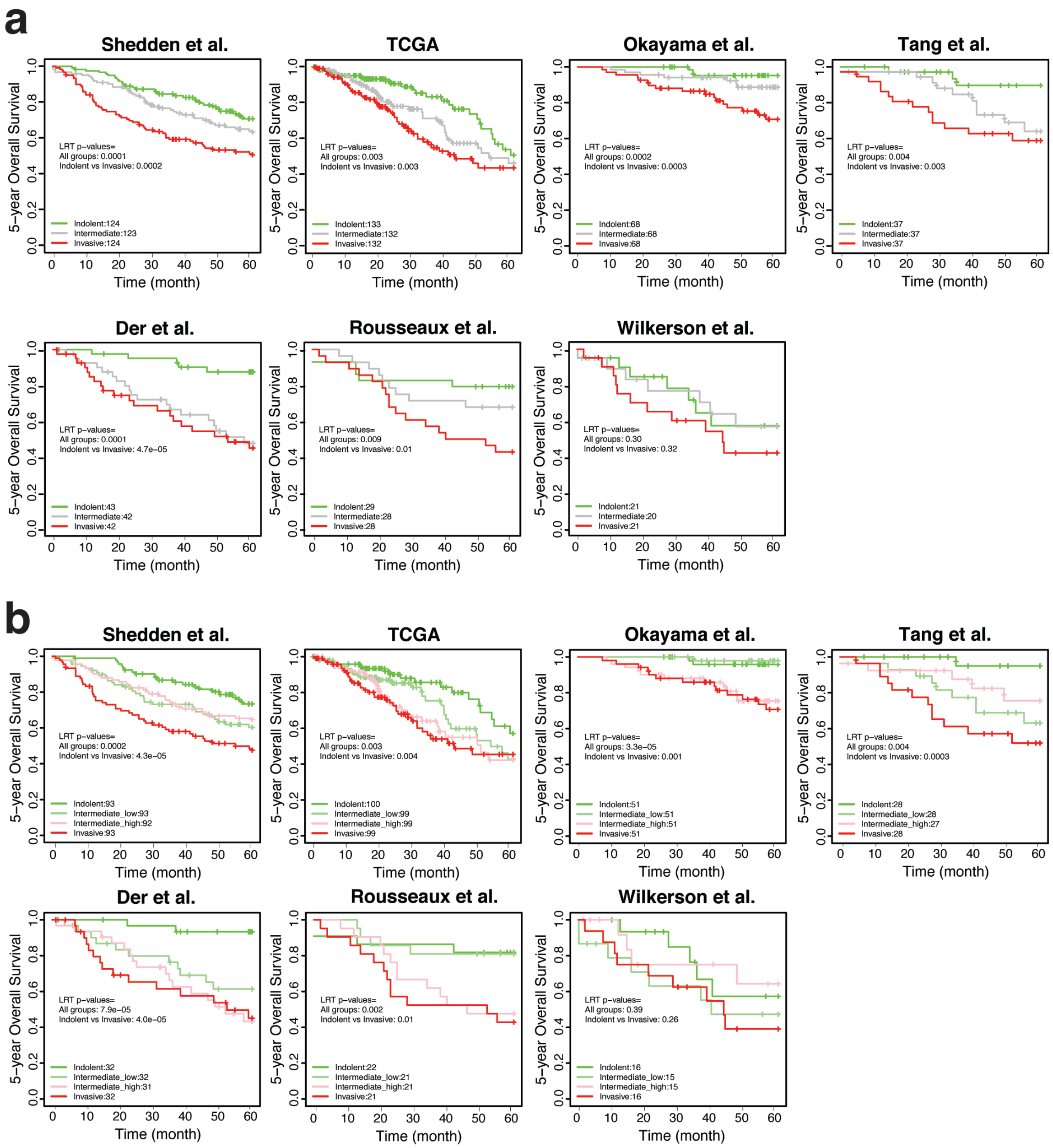

Supplementary Fig 3. Survival differences among patients grouped by IVS.

a. The number of patients were evenly distributed into 3 groups. The indolent group with the lowest IVS is marked in red and the invasive group with the highest IVS is shown in green. Source data are provided in Source Data files.

b. The number of patients were evenly distributed into 4 groups.

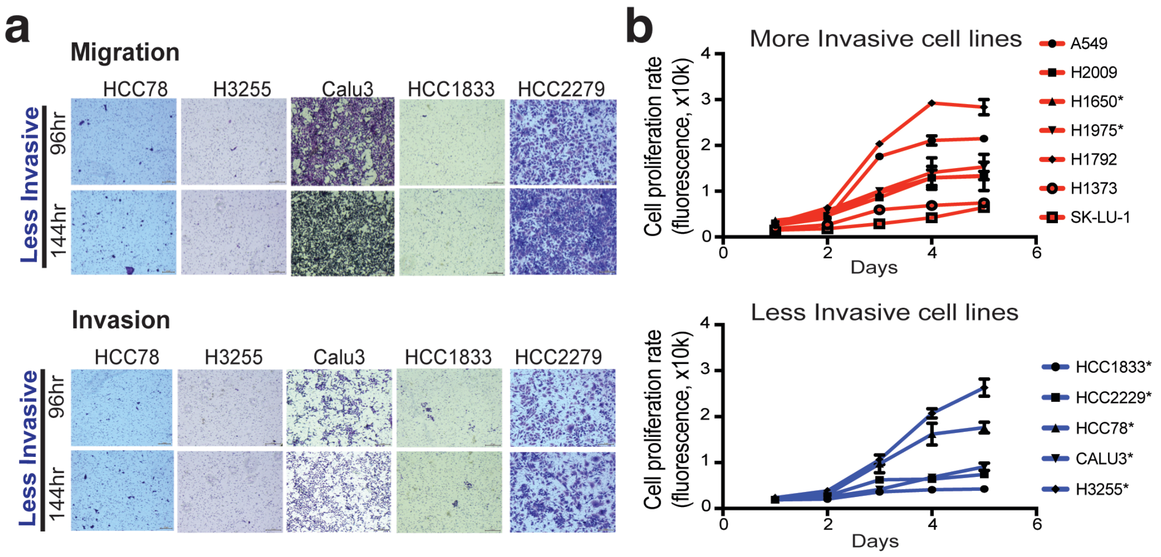

Supplementary Fig. 4. Migration, invasion, and proliferation assay LUAD cells.

a. Representative images of migrated and invaded cells in transwell migration and transwell matrigel invasion assay after 96hr and 144hr in 5 less invasive LUAD cells. Scale bar 10µm.

b. Cell proliferation rate for 7 more invasive LUAD cell lines (top) and 5 less invasive cell lines (bottom) from day1 to day5 using alamar blue assay. KRAS wild type cells are marked with *. n=3. Data presented as mean ± s.e.m. Data points were connected through connecting lines. Source data are provided in Source Data files.

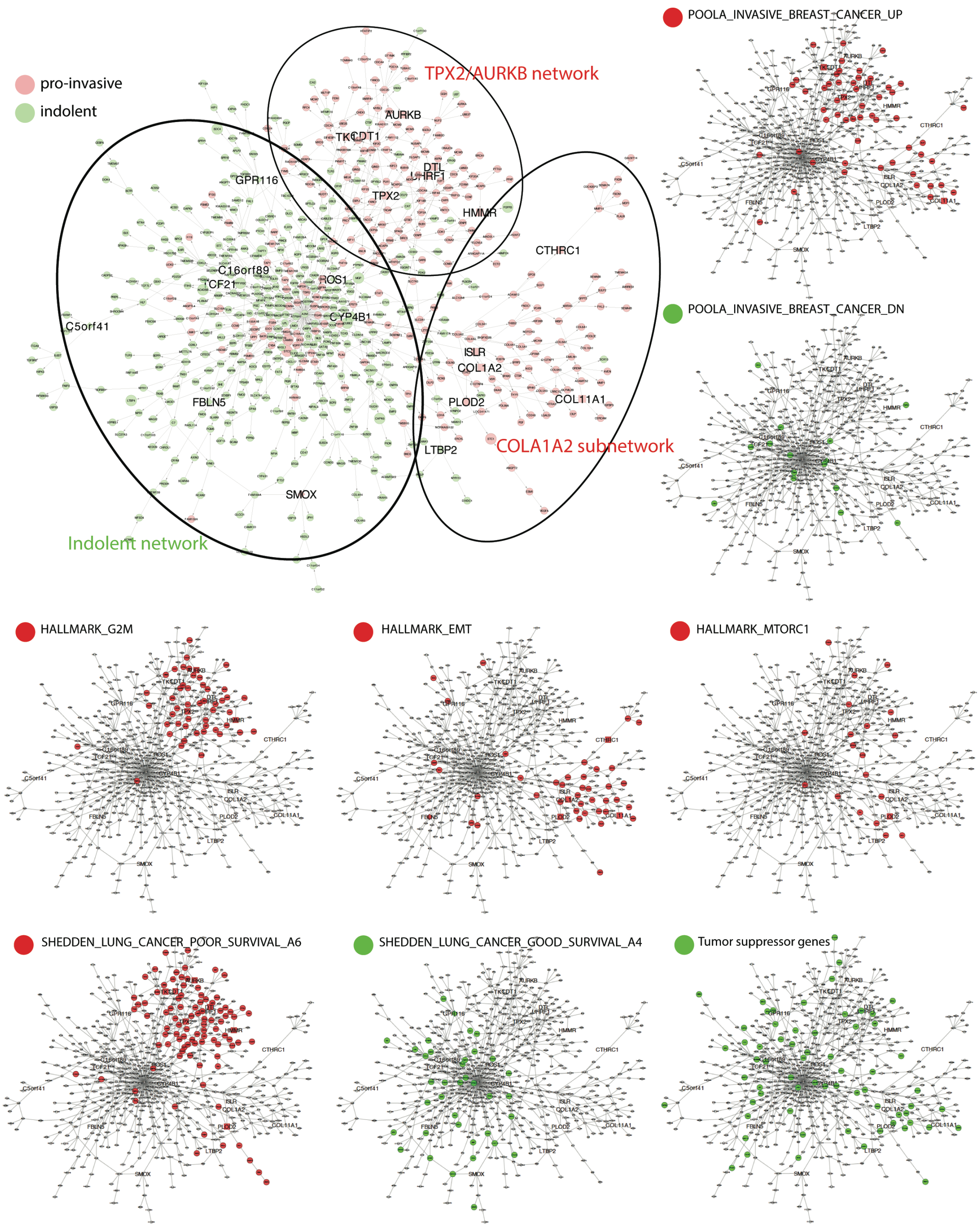

Supplementary Fig. 5. Integrative regulatory network for early-stage lung adenocarcinoma constructed using TCGA LUAD stage I tumors.

Subnetwork focusing on pro-invasive and indolent signature genes. Nodes are colored for the pro-invasive (red) and indolent (green) signature genes on the top-left corner. Genes from the selected

gene sets from Figure 1d are projected on the network and colored according to their association with the tumor invasion. Source data are provided in Source Data files.

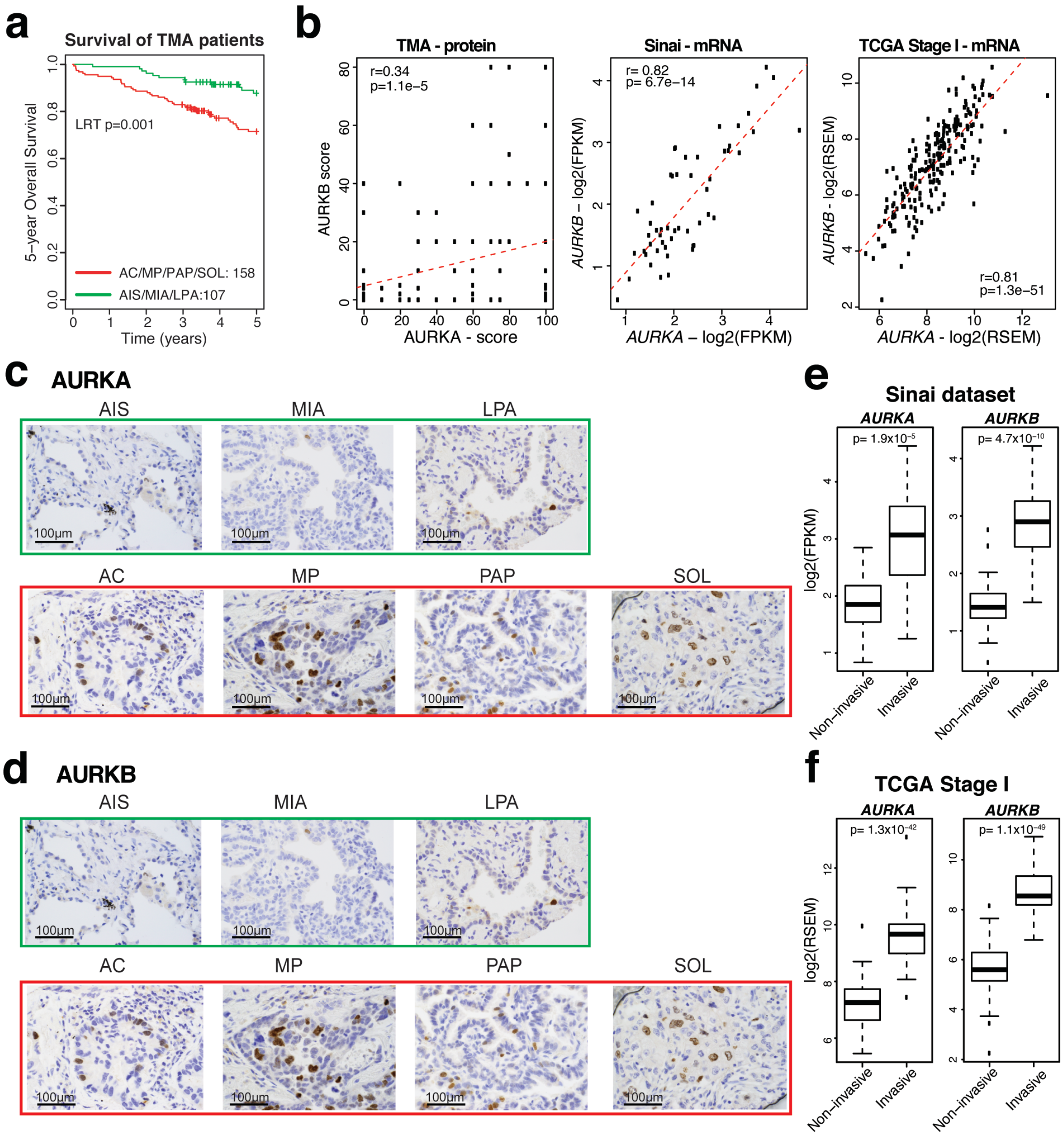

Supplementary Fig. 6. AurA and AurB expression between invasive and non-invasive tumors

A. Survival difference between invasive and non-invasive tumors in stage I TMA data. LRT p-value was measured. Source data are provided in Source Data files.

B. AurA and AurB protein expression from stage I tumor in the TMA dataset (left), mRNA expression across 53 samples from our original dataset (Sinai) (middle), and mRNA expression across 216 TCGA Stage 1 RNAseq samples (right). Pearson correlation coefficient and two-sided test p-values were estimated. Source data are provided in Source Data files.

C. Representative images from immunohistochemical staining of AurA in AIS, MIA, LPA vs AC, MP, PAP and SOL in human TMA. Scale bar 100µm.

D. Representative images from immunohistochemical staining of AurB in AIS, MIA, LPA vs AC, MP, PAP and SOL in human TMA. Scale bar 100µm.

E. Comparison of mRNA levels of *AURKA* and *AURKB* between invasive (n=21) and non-invasive (n=32) tumors from the Sinai dataset. The middle line of the box is median values for each group and the box edges are the 25th and 75th percentiles. Whiskers indicate maximum and minimum values of each group except for outliers. Two-side t-test p-value was measured.

F. Comparison of mRNA levels of *AURKA* and *AURKB* between invasive (n=55) and non-invasive (n=118) tumors from the TCGA Stage I samples. The middle line of the box is median values for each group and the box edges are the 25th and 75th percentiles. Whiskers indicate maximum and minimum values of each group except for outliers. Two-side t-test p-value was measured.

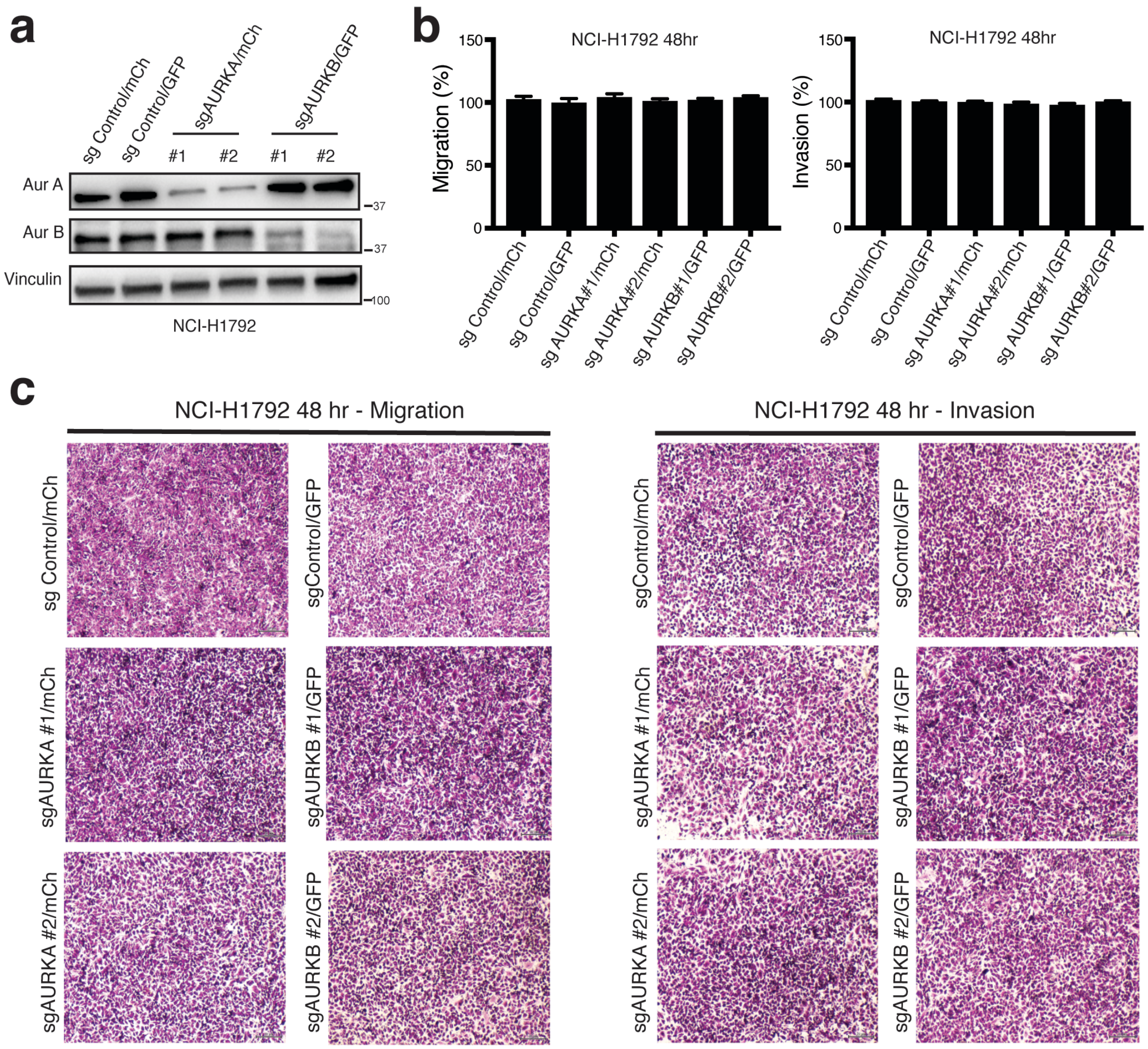

Supplementary Fig. 7. CRISPR deletion of aurora kinase A or -B alone has no effect on migration and invasion in LUAD cells.

- **a.** Western blot for H1792 cells transduced with indicated sgRNAs.
- **b.** Quantitation of percent migration and invasion of H1792 cells transduced with indicated sgRNAs at 48hr. Data presented as mean ± s.e.m. No significant difference was observed for migration and invasion assay for all comparisons (n=4). Source data are provided in Source Data files.
- **c.** Representative images of migrated and invaded cells in transwell assay for H1792 transduced with indicated sgRNAs. Scale bar 10μm.

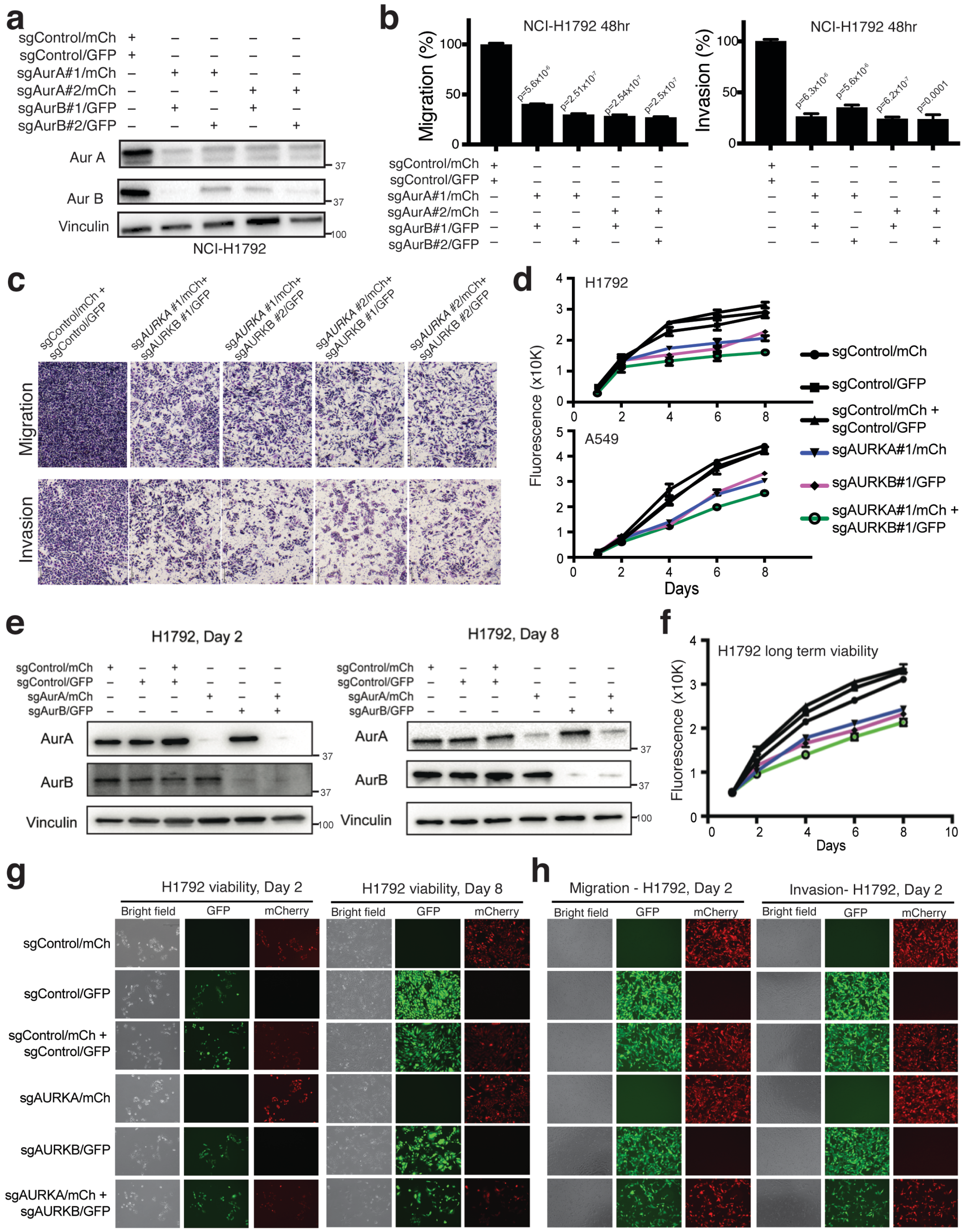

Supplementary Fig. 8. CRISPR deletion of both aurora kinase A and B suppress migration and invasion in LUAD cells.

a. Western blot for H1792 cells transduced with indicated sgRNAs.

b. Quantitation of percent migration and invasion of H1792 cells transduced with indicated sgRNAs at 48hr. Data presented as mean ± s.e.m. Two-side t-test p-values with respect to control are provided (n=4). Source data are provided in Source Data files.

c. Representative images of migrated and invaded cells in transwell assay for H1792 transduced with indicated sgRNAs at 48hr. Scale bar 10µm.

d. Cell Proliferation shown as fluorescence measured by alamar blue assay (normalized to Day 1) showing effect of indicated sgRNA in (left) H1792 and (right) A549 cells from day 1 to Day 8. n=3. Data presented as mean ± s.e.m. Data points were connected through connecting lines. Source data are provided in Source Data files.

e. Western blot for H1792 cells transduced with indicated sgRNAs at day 2 and day8.

f. Cell Proliferation shown as fluorescence measured by alamar blue assay (normalized to Day 1) showing effect of indicated sgRNA in H1792 cells from day 1 to Day 8. n=3. Colors of each condition is same as in panel d. Source data are provided in Source Data files.

g. Bright field and fluorescence images to visualize viability of cells transduced with indicated sgRNAs at Day 2 and day 8.

h. Bright field and fluorescence images for migration and invasion of cells transduced with indicated sgRNAs at Day2.

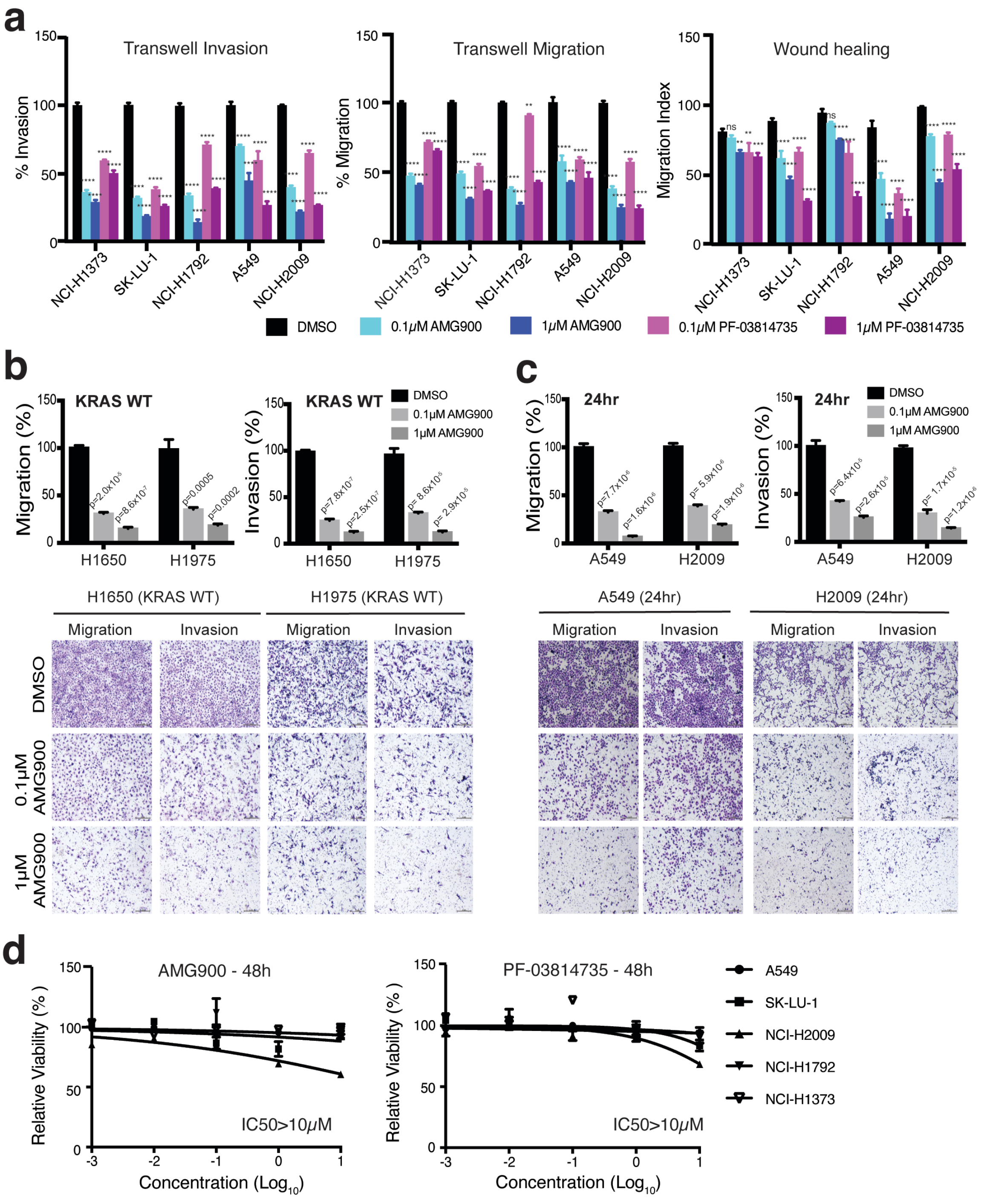

Supplementary Fig. 9. Pan aurora kinase inhibitors suppress migration and invasion in panel of invasive LUAD cells via suppressing aurora kinases activity.

a. Quantification of %Migration, %Invasion from transwell migration and invasion assays respectively, and migration index from wound healing assay, for panel of 5 invasive LUAD

cells treated with DMSO, AMG900 (0.1μM, 1μM) and PF-03814735 (0.1μM, 1μM). For migration and invasion assay, n=8 for DMSO and n=4 for AMG900 (0.1μM, 1μM) and PF-03814735 (0.1μM, 1μM). For wound healing assay n=16 for DMSO and n=8 for AMG900 (0.1μM, 1μM) and PF-03814735 (0.1μM, 1μM). Data presented as mean ± s.e.m. Significant comparison from two-side t-test is marked with asterisk. Exact p-value for each test is provided in Supplementary Table 7. Source data are provided in Source Data files.

b. Quantification (top) of %Migration and %Invasion from transwell migration and invasion assays in 2 KRAS wild type highly invasive cell lines H1650 and H1975 treated with DMSO and AMG900 (0.1μM, 1μM). Representative images are shown at bottom, Scale bar 10μm. Data presented as mean ± s.e.m. Two-side t-test p-values are provided (n=3). Source data are provided in Source Data files.

c. Quantification (top) of %Migration and %Invasion from transwell migration and invasion assays in 2 highly invasive cell lines A549 and H2009 treated with DMSO and AMG900 (0.1μM, 1μM) measured at 24hr (timeframe shorter than their doubling time). Representative images are shown at bottom, Scale bar 10μm. Data presented as mean ± s.e.m. Two-side t-test p-values are provided (n=3). Source data are provided in Source Data files.

d. Relative viability of panel of 5 invasive LUAD cells treated with DMSO vs serial dilution of AMG900 or PF-03814735 for 48hr. log(inhibitor) vs. normalized response -- Variable slope, n=3, Data presented as mean ±s.e.m. Source data are provided in Source Data files.

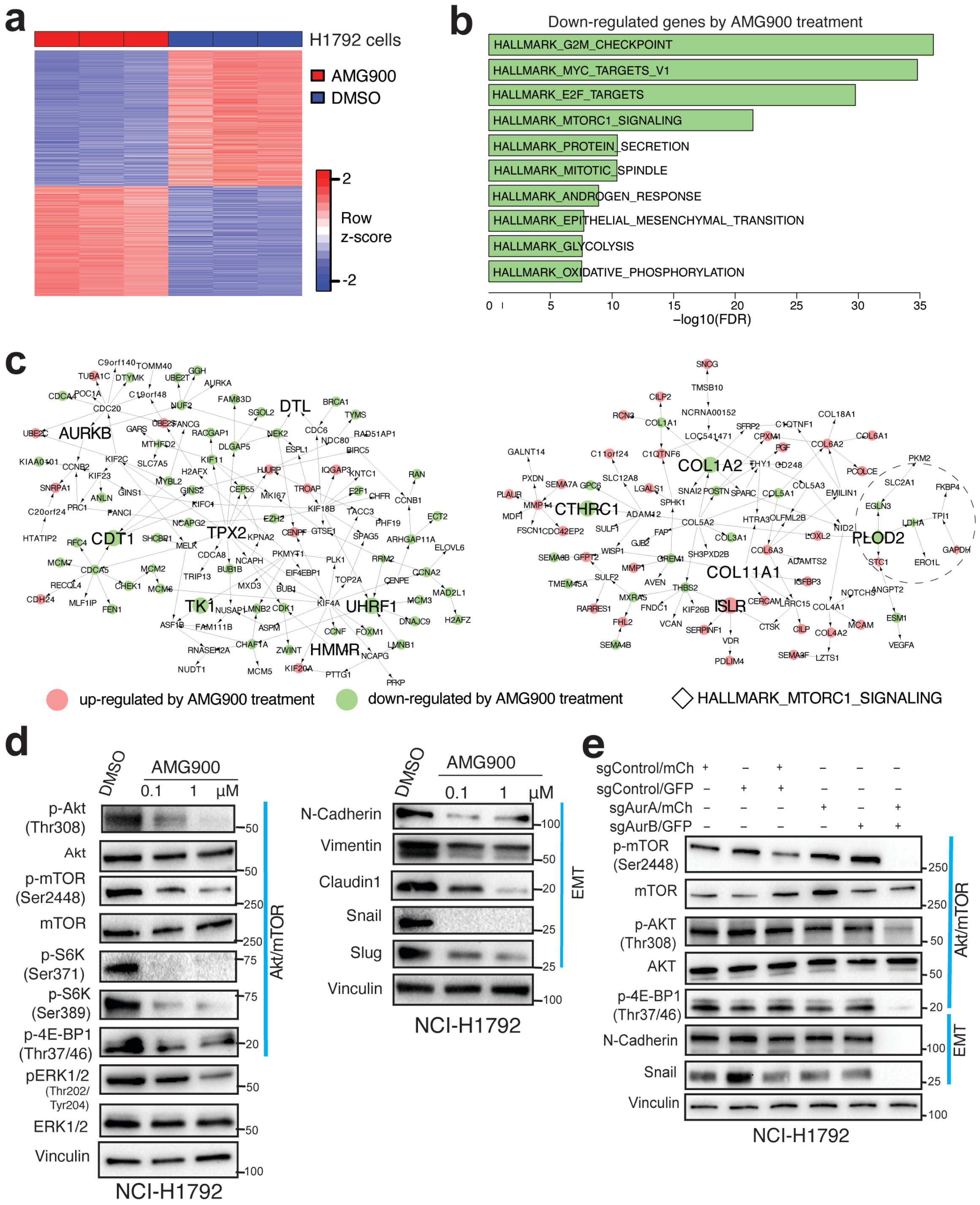

Supplementary Fig. 10. Aurora kinases drive invasiveness in lung adenocarcinoma through activating AKT/mTOR and EMT pathways

- **a.** Differentially expressed genes between DMSO and 0.1μM AMG900 treated H1792 cells.
- **b.** Top 10 down-regulated hallmark pathways by AMG900 treatment.

c. Overlaying the DEGs onto the TPX2/AURKB and COL1A2 subnetworks. Nodes filled in red are up-regulated and ones in green are down-regulated by AMG900 treatment. Genes included in HALLMARK_MTORC1_SIGLANING pathways are indicated with diamond shapes (□).

d. Western blot for indicated proteins in H1792 cells treated with DMSO and indicated concentrations of AMG900 for 48hr.

e. Western blot for H1792 cells treated with indicated sgRNAs for AKT/mTOR and EMT pathway.

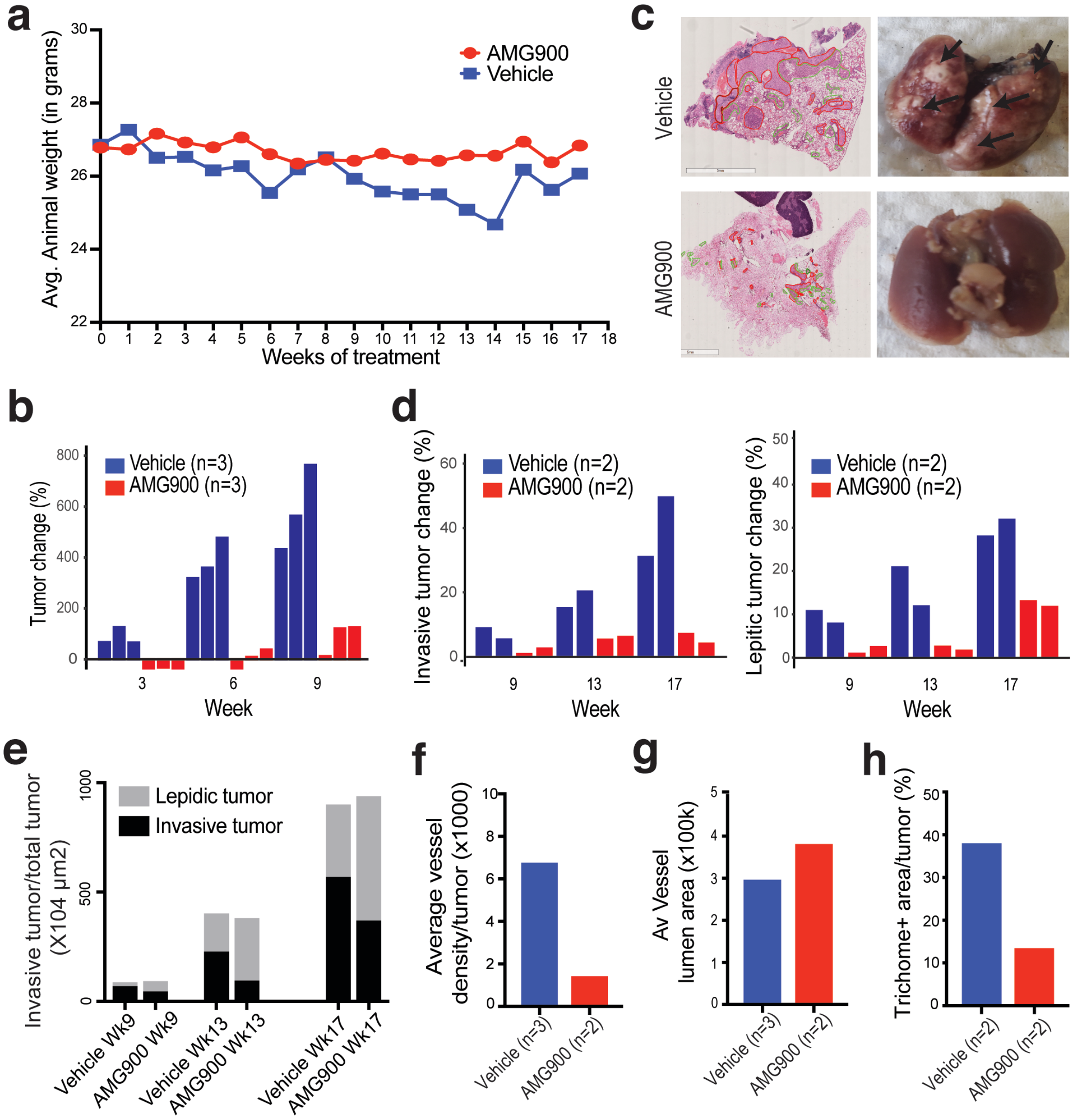

Supplementary Fig. 11. Aurora kinase inhibition suppresses progression of invasive LUAD in Kras(G12D)/TGFBR2$^{-/-}$ mouse model

a. Average animal weight for vehicle and AMG900 treated animals after treatment starting till week 17. Drop in animal weight in vehicle group reflects loss in body weight of sick animal, which died at that time point. Vehicle n=8, AMG900 n=10. Source data are provided in Source Data files.

b. Waterfall plot showing percent change in tumor burden from micro-CT data for vehicle and AMG900 treated animals. (n=3). Source data are provided in Source Data files.

c. (left) Representative images showing annotated invasive and lepidic tumor areas in vehicle (Scale bar 3mm) and AMG900 (Scale bar 5mm) treated animal's lung. (right) photographs of dissected lungs from vehicle and AMG900 treated animals. Several tumor nodules (arrows) are seen by naked eye in vehicle treated animal lung.

d. Waterfall plot showing percent change in invasive and lepidic tumor areas from histopathological analysis for vehicle and AMG900 treated animals. (n=2)

e. Stacked plot showing invasive tumor lesion area per total tumor area in vehicle and AMG900 treated animals with equal overall tumor burden at week 9, 13 and 17. (n=2). Source data are provided in Source Data files.

f. Average vessel density from CD31 IHC staining for tumor infiltrating neovessels in in vehicle and AMG900 treated animal's lung. (vehicle n=3, AMG900 n=2) Source data are provided in Source Data files.

g. Average vessel lumen area quantified from CD31 IHC staining in vehicle and AMG900 treated animal's lung. Most vessels in the vehicle group tumor appeared collapsed while in AMG900 treated mouse tumor vessels were wider, however the difference wasn't significant between both groups. (vehicle n=3, AMG900 n=2) Source data are provided in Source Data files.

h. Quantification of collagen deposition from Masson's trichome staining in vehicle and AMG900 treated animal's lung. (n=2) Source data are provided in Source Data files.

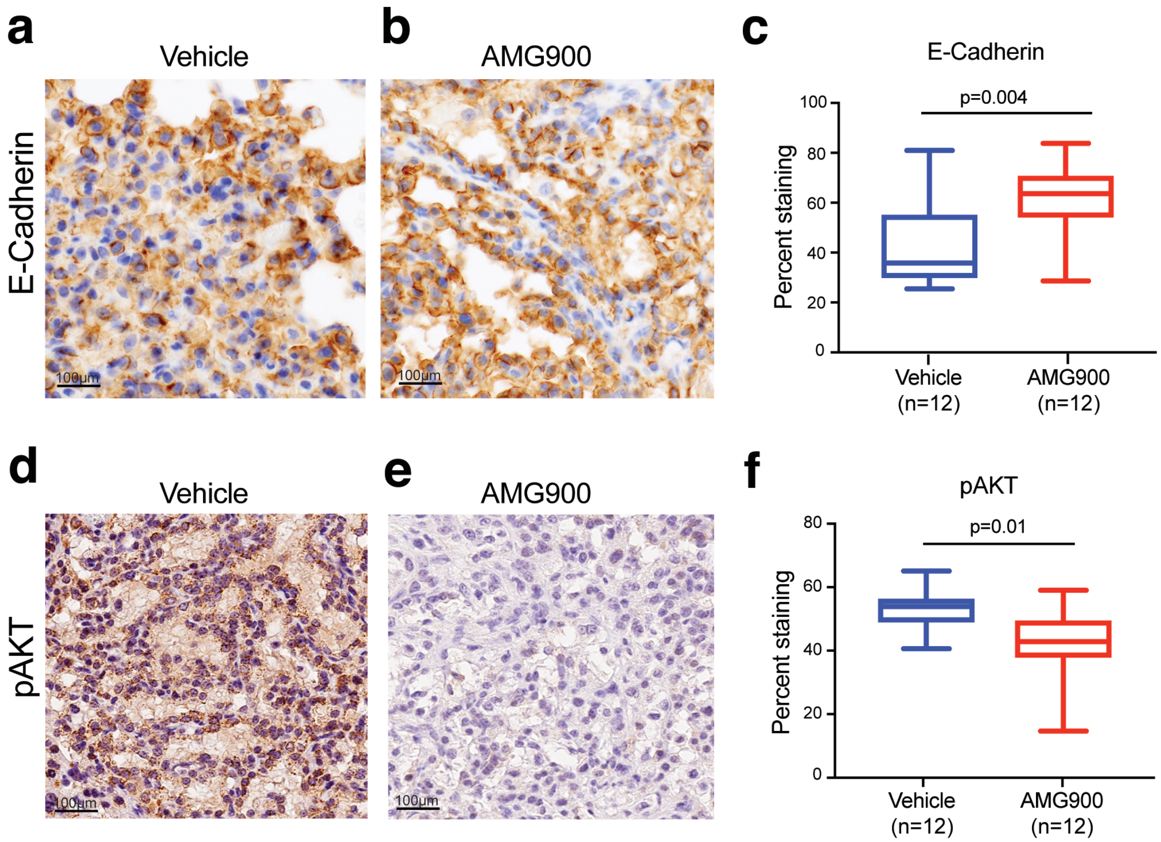

Supplementary Fig. 12. Aurora kinase inhibition leads to upregulation in EMT marker E-Cadherin and suppression of pAKT in Kras(G12D)/TGFBR2$^{-/-}$ mouse model

(a-b) E-Cadherin immunostaining showing reduction in staining intensity in transition to invasive growth pattern in (a) vehicle controls when compared to uniform strong membranous staining in (b) AMG900 treated mice. Scale bar 100µm.

(c) Box plot shows values of twelve regions of interest for E-Cadherin staining analyzed by Image J plugin IHC profiler. Center line represents the median value (50th percentile), while the box contains the 25th to 75th percentiles of dataset. The whiskers mark the minimum and maximum values. Two-side t-test p=0.004 without multiple correction, n=12 (4 regions analyzed from 3 mice). Source data are provided in Source Data files.

(d-e) Immunohistochemistry for pAKT showing strong staining in tumors cells in (d) vehicle animals while significantly low staining in (e) AMG900 treated mice. Scale bar 100µm.

(f) Box plot shows values of twelve regions of interest for pAKT staining analyzed by Image J plugin IHC profiler. Center line represents the median value (50th percentile), while the box contains the 25th to 75th percentiles of dataset. The whiskers mark the minimum and maximum values, respectively. Two-side t-test p=0.01 without multiple correction, n=12 (4 regions analyzed from 3 mice). Source data are provided in Source Data files. (a, b, d. e, Original magnification x150).

Supplementary Tables

STable 1. Clinical information of 53 early-stage lung adenocarcinoma

CaseID	PRED.HISTO	T.STAGE	N.STAGE	Subgroup
NIHAD1	MP	T1A	N1	Invasive
NIHAD10	MP	T1A	N1	Invasive
NIHAD11	AC	T1A	N0	Non-invasive
NIHAD14	MP	T1A	N0	Invasive
NIHAD15	MP	T1A	N0	Invasive
NIHAD18	SOL	T1A	N0	Invasive
NIHAD20	SOL	T1A	N0	Invasive
NIHAD21	LPA	T1A	N0	Non-invasive
NIHAD25	LPA	T1A	N0	Non-invasive
NIHAD28	LPA	T1A	N0	Non-invasive
NIHAD29	LPA	T2A	N0	Non-invasive
NIHAD30	LPA	T1A	N0	Non-invasive
NIHAD31	MIA	T1A	N0	Non-invasive
NIHAD32	LPA	T1B	N0	Non-invasive
NIHAD35	MIA	T1A	NX	Non-invasive
NIHAD36	AIS	T1A	N0	Non-invasive
NIHAD37	AIS	T1A	N0	Non-invasive
NIHAD38	AIS	T1A	N0	Non-invasive
NIHAD4	SOL	T1A	N1	Invasive
NIHAD40	AIS	T1A	N0	Non-invasive
NIHAD44	AIS	T1B	N0	Non-invasive
NIHAD45	MIA	T1A	N0	Non-invasive
NIHAD47	MIA	T1A	N0	Non-invasive
NIHAD48	AIS	T1A	N0	Non-invasive
NIHAD49	MIA	T1A	N0	Non-invasive
NIHAD50	MIA	T2A	N0	Non-invasive
NIHAD51	MP	T1B	N0	Invasive
NIHAD52	SOL	T1A	N0	Invasive
NIHAD53	AC	T1A	N0	Non-invasive
NIHAD58	AC	T1B	N0	Invasive
NIHAD61	AC	T1B	N2	Invasive
NIHAD62	AC	T1B	N1	Invasive
NIHAD63				Invasive
NIHAD64	PAP	T1B	N2	Non-invasive

NIHAD66	SOL	T1B	N2	Invasive
NIHAD67	AC	T1B	N1	Invasive
NIHAD68	PAP	T1B	N0	Invasive
NIHAD69	LPA	T1B	N0	Invasive
NIHAD70	LPA	T2A	N0	Non-invasive
NIHAD71	LPA	T1B	N0	Non-invasive
NIHAD73	LPA	T1A	N0	Non-invasive
NIHAD74	LPA	T2A	N0	Non-invasive
NIHAD75	LPA	T1A	N0	Non-invasive
NIHAD77	LPA	T1B	N0	Invasive
NIHAD8	SOL	T1A	N1	Invasive
NIHAD80	AIS	T1A	N0	Non-invasive
NIHAD81	AIS	T1B	N0	Invasive
NIHAD82	AIS	T1A	N0	Non-invasive
NIHAD83	AIS	T1B	N0	Non-invasive
NIHAD85	AIS	T2A	N0	Non-invasive
NIHAD87	AIS	T1A	N0	Non-invasive
NIHAD88	AIS	T1A	N0	Non-invasive
NIHAD9	SOL	T1A	N1	Invasive

STable 2. Comparison of invasive signatures (pro-invasive and indolence signatures) with DEGs from mouse model

	Mouse model (Tgfbr2 status)	
	Upregulated in Tgfbr2 -/- (n=364)	Upregulated in Tgfbr2 WT (n=316)
Pro-invasive (n=456)	*1810055G02Rik, Aldoa, Angptl4, Cd248, Cda, Col5a2, Col7a1, Fam20c, Fhl2, Gjb3, Gpc1, Gpc6, Gtse1, Htra3, Igfbp3, Kpna2, Mmp11, Mmp14, Mt2, Nid2, Olfml2b, Plek2, Psrc1, S100a16, Sdcbp2, Slc6a8, Spp1, Srpx2, Thbs2, Timp1, Tpi1, Uck2, Wisp1* (33, OR=4.97, p=1.6e-12)	*Ido1, Tap2* (2, OR=0.30, p=0.99)
Indolence (n=683)	*AA986860, Aqp5, Bcam, Cldn18, Clic5, Epb4.1l4a, Epha3, Ephx2, Fam189a2, Flrt3, Gdf15, Icam4, Itga9, Kcnk5, Megf11, Ndnf, Pard6b, Prg4, Prss16, Pvrl3, Scara3, Tgfb2, Tmem98, Zfp458* (24, OR=2.23, p=0.0005)	*Aff3, Angpt1, Ankrd29, Bmp5, Camk1d, Cass4, Cd207, Cldn1, Esyt3, Fmo2, Fmo5, Hlf, Limd1, Lztfl1, Mamdc2, Per3, Scube2, Slc46a3, Tppp, Zfp874a* (20, OR=2.12, p=0.002)

STable 3. Comparison of survival association of tumor groups classified with different IVS brackets

Invasive vs Indolent	Local_minima		Fixed IVS value cutoff					
			IVS>0.95 vs IVS<0.05		IVS>0.9 vs IVS<0.1		IVS>0.8 vs IVS<0.2	
	HR(95% CI)	LRT p-value	HR(95% CI)	LRT p-value	HR(95% CI)	LRT p-value	HR(95% CI)	LRT p-value
Shedden et al. (n=371)	2.62(1.65-4.17)	8.30E-05	3.99(2.27-7.00)	3.70E-06	3.01(1.85-4.88)	1.30E-05	2.12(1.39-3.24)	5.00E-04
TCGA (n=397)	2.37(1.34-4.19)	0.004	2.07(1.20-3.57)	0.009	2.24(1.38-3.64)	0.001	1.63(1.05-2.52)	0.03
Okayama et al. (n=204)	9.01(2.48-32.79)	0.0002	7.82(2.15-28.43)	0.0005	9.86(2.81-34.61)	2.80E-05	8.69(2.86-26.42)	1.68E-05
Tang et al. (n=111)	7.31(1.47-36.25)	0.007	5.81(1.06-31.75)	0.04	7.56(2.08-27.50)	0.0006	3.19(1.26-8.11)	0.01
Der et al. (n=127)	5.82(2.09-16.22)	0.0003	13.92(3.10-62.49)	1.70E-05	6.23(2.30-16.84)	5.00E-05	5.09(2.15-12.01)	4.94E-05
Rousseaux et al. (n=85)	3.17(1.15-8.76)	0.02	5.13(1.60-16.49)	0.003	3.21(1.21-8.47)	0.01	3.51(1.45-8.50)	0.003
Wilkerson et al. (n=62)	3.30(1.07-10.20)	0.05	1.34(0.56-3.20)	0.52	1.37(0.61-3.07)	0.45	1.11(0.54-2.26)	0.79

Invasive vs Indolent	Fixed IVS percentile cutoff							
	Top 5% vs Bottom 5%		Top 10% vs Bottom 10%		Top 20% vs Bottom 20%		Top 30% vs Bottom 30%	
	HR(95% CI)	LRT p-value	HR(95% CI)	LRT p-value	HR(95% CI)	LRT p-value	HR(95% CI)	LRT p-value
Shedden et al. (n=371)	3.76(0.99-14.19)	4.00E-02	5.24(2.23-12.32)	2.91E-05	3.24(1.81-5.80)	2.86E-05	2.52(1.58-4.04)	6.71E-05
TCGA (n=397)	9.31(1.19-72.78)	0.005	2.70(1.12-6.51)	0.02	2.74(1.46-5.14)	1.00E-03	1.95(1.19-3.20)	0.007
Okayama et al. (n=204)	-	0.09	-	0.01	6.02(1.32-27.53)	0.007	10.17(2.35-44.07)	5.25E-05
Tang et al. (n=111)	-	0.01	-	0.007	12.78(1.65-99.10)	0.0008	7.42(1.67-32.89)	0.001
Der et al. (n=127)	10.87(1.02-115.51)	0.03	12.69(1.49-107.72)	0.004	9.11(2.03-40.85)	0.0004	14.64(3.40-63.06)	1.01E-06
Rousseaux et al. (n=85)	-	0.03	-	0.01	6.12(1.34-28.07)	0.006	3.13(1.22-8.04)	0.01
Wilkerson et al. (n=62)	3.10(0.55-17.42)	0.18	1.07(0.51-2.25)	0.18	1.75(0.72-4.27)	0.21	1.07(0.51-2.25)	0.86

"-" : Hazard ratios are not converged mainly due to small number of samples

Invasive vs Indolent	Even number of samples in each group			
	Top vs Bottom out of 4 groups		Top vs Bottom out of 3 groups	
	HR(95% CI)	LRT p-value	HR(95% CI)	LRT p-value
Shedden et al. (n=371)	2.82(1.68-4.74)	4.30E-05	2.28(1.47-3.55)	0.0002
TCGA (n=397)	2.26(1.28-4.00)	0.004	2.05(1.27-3.31)	0.003
Okayama et al. (n=204)	7.57(1.71-33.56)	0.001	6.67(1.95-22.79)	0.0003
Tang et al. (n=111)	14.09(1.83-108.43)	0.0003	5.21(1.50-18.14)	0.003
Der et al. (n=127)	11.12(2.54-48.80)	4.00E-05	5.99(2.24-16.01)	4.70E-05
Rousseaux et al. (n=85)	3.74(1.20-11.65)	0.01	3.19(1.24-8.18)	0.01
Wilkerson et al. (n=62)	1.86(0.62-5.55)	0.26	1.61(0.62-4.16)	0.32

STable 4. Key drivers for the signature genes

For pro-invasive signatures					For indolence signatures				
**GeneName**	Target	Overlap	OR	p-value	**GeneName**	Target	Overlap	OR	p-value
TPX2	79	42	24.51	1.06E-34	***C16orf89***	120	47	9.34	2.79E-24
COL1A2	34	24	49.4	1.60E-24	***GPR116***	32	22	30.64	7.36E-19
AURKB	36	23	36.31	5.30E-22	***TCF21***	34	21	22.45	1.30E-16
UHRF1	34	22	37.53	2.83E-21	***SMOX***	26	17	26.05	2.79E-14
CDT1	28	17	31.25	5.17E-16	***ROS1***	32	18	17.75	2.04E-13
COL11A1	18	14	70.15	9.60E-16	***CYP4B1***	794	109	2.41	5.33E-13
CTHRC1	23	13	25.99	5.76E-12	***C5orf41***	26	14	16	2.29E-10
TK1	19	11	27.35	1.84E-10	***FBLN5***	13	10	45.4	5.71E-10
ISLR	27	12	15.94	1.47E-09	***LTBP2***	34	15	10.84	1.89E-09

ROR2	22	11	19.89	1.50E-09
PLOD2	16	9	25.44	1.27E-08
HMMR	12	8	39.48	1.31E-08
DTL	13	8	31.58	3.25E-08

STable 5. Gene set enriched within TPX2/AURKB and COL1A2 subnetworks

TPX2/AURKB subnetwork

Geneset	Size	Overlap	OddRatio	p.value
HALLMARK_G2M_CHECKPOINT	131	49	66.93	1.45E-59
HALLMARK_E2F_TARGETS	146	49	56.50	8.71E-57
HALLMARK_MITOTIC_SPINDLE	159	25	15.74	1.50E-19
HALLMARK_SPERMATOGENESIS	43	10	22.42	3.44E-10
HALLMARK_MYC_TARGETS_V1	116	13	9.52	9.62E-09
HALLMARK_MTORC1_SIGNALING	159	10	4.90	9.37E-05
SHEDDEN_LUNG_CANCER_POOR_SURVIVAL_A6	303	83	76.91	3.82E-94
POOLA_INVASIVE_BREAST_CANCER_UP	218	36	18.68	5.73E-29
PUIFFE_INVASION_INHIBITED_BY_ASCITES_UP	57	8	11.85	1.36E-06
WINNEPENNINCKX_MELANOMA_METASTASIS_UP	112	46	75.25	5.09E-58
BIDUS_METASTASIS_UP	127	28	24.70	3.76E-26
SUNG_METASTASIS_STROMA_DN	36	9	24.47	1.36E-09
JAEGER_METASTASIS_UP	31	8	25.32	8.86E-09
WANG_METASTASIS_OF_BREAST_CANCER_ESR1_UP	16	6	42.95	5.65E-08
LIAO_METASTASIS	389	18	3.71	1.04E-05
SARRIO_EPITHELIAL_MESENCHYMAL_TRANSITION_UP	135	45	53.12	1.02E-51

COL1A2 subnetwork

Geneset	Size	Overlap	OddRatio	p.value
HALLMARK_EPITHELIAL_MESENCHYMAL_TRANSITION	168	34	33.36	1.85E-34
HALLMARK_GLYCOLYSIS	156	12	8.12	1.70E-07
HALLMARK_HYPOXIA	157	12	8.07	1.83E-07
HALLMARK_ANGIOGENESIS	28	6	25.16	5.68E-07
HALLMARK_MYOGENESIS	121	9	7.60	8.62E-06
HALLMARK_UV_RESPONSE_DN	119	7	5.78	0.000391674
HALLMARK_MTORC1_SIGNALING	159	8	4.93	0.000432202
ANASTASSIOU_MULTICANCER_INVASIVENESS_SIGNATURE	56	28	121.08	3.06E-41
SCHUETZ_BREAST_CANCER_DUCTAL_INVASIVE_UP	305	38	19.68	2.17E-30

POOLA_INVASIVE_BREAST_CANCER_UP	218	15	7.42	1.93E-08
CLASPER_LYMPHATIC_VESSELS_DURING_METASTASIS_DN	30	9	40.86	2.55E-11
SUNG_METASTASIS_STROMA_UP	91	9	10.41	7.91E-07

STable 6. Demographics of TMA patients

**Sample size (count)**	396
**Age (median + sd)**	67.3±9.7
**Gender**	Female (186), Male (87), Unknown (123)
**Histological subtype**	MIA(64), AIS(43), LPA(24), MP(14), PAP(56), AC(153), SOL(42)
**# of patients with AURKASCORE**	369
**# of patients with AURKBSCORE**	169

STable 7. Statistical tests of phenotype changes with aurora kinase inhibitions

Linear models dose dependent effect of aurora kinase inhibition for Figure 4e

		Invasion assay		Migration assay		Wound healing assay	
Drug	Cell line	t-value	p-value	t-value	p-value	t-value	p-value
AMG900	NCI-H1373	-2.69	0.0226	-2.74	0.0209	-3.67	0.0006
	SK-LU-1	-3.04	0.0125	-3.72	0.0040	-9.83	7.04E-13
	NCI-H1792	-3.44	0.0063	-3.10	0.0112	-5.46	1.87E-06
	A549	-4.80	0.0007	-3.34	0.0075	-5.66	1.07E-05
	NCI-H2009	-3.50	0.0058	-3.36	0.0072	-14.57	7.52E-19
PF-03814735	NCI-H1373	-3.06	0.0121	-3.08	0.0117	-1.46	0.1584
	SK-LU-1	-3.02	0.0130	-3.86	0.0032	-11.36	1.13E-10
	NCI-H1792	-7.07	3.42E-05	-33.50	1.33E-11	-5.56	1.37E-05
	A549	-5.26	0.0004	-3.28	0.0082	-3.29	0.008
	NCI-H2009	-7.18	3.01E-05	-5.64	0.0002	-8.63	1.64E-08

Two-side t-test for Supplementary Figure 9a

		Invasion assay		Migration assay		Wound healing assay	
Drug	Cell line	0.1uM	1uM	0.1uM	1uM	0.1uM	1uM
AMG900	NCI-H1373	1E-07	8.5E-08	2E-07	1.6E-08	0.0019	2.068E-06
	SK-LU-1	9E-08	2.3E-08	7E-07	3.6E-08	0.0003	5.9058E-09

	NCI-H1792	5E-06	1.5E-06	2E-08	2.4E-09	4E-09	1.3601E-12
	A549	0.0019	0.00044	0.0016	0.00012	0.0021	0.00010145
	NCI-H2009	1E-08	4E-10	1E-06	9.7E-08	3E-09	8.9101E-14
	NCI-H1373	6E-05	3.2E-05	5E-06	1.7E-06	0.254	0.00231303
	SK-LU-1	4E-06	8.8E-07	5E-07	5E-09	0.0004	7.4758E-11
	NCI-H1792	6E-05	1.2E-07	0.0007	1.7E-08	0.0291	3.0711E-07
	A549	0.001	3.2E-07	0.0003	0.00016	0.0021	0.00053798
PF-03814735	NCI-H2009	3E-06	2.9E-10	2E-05	1.4E-07	1E-08	1.7689E-08

STable 8. HALLMARK pathways enriched in down-regulated genes by AMG900 treatment

A549 cells

Geneset	Size	Overlap	OddRatio	p-value	q-value
HALLMARK_E2F_TARGETS	194	168	42.94	1.28E-116	6.38E-115
HALLMARK_G2M_CHECKPOINT	191	152	25.72	1.45E-94	3.63E-93
HALLMARK_MYC_TARGETS_V1	193	124	11.71	3.30E-59	5.49E-58
HALLMARK_MITOTIC_SPINDLE	198	100	6.57	4.40E-35	5.50E-34
HALLMARK_MTORC1_SIGNALING	195	79	4.35	2.50E-20	2.50E-19
HALLMARK_MYC_TARGETS_V2	58	31	7.23	1.12E-12	9.30E-12
HALLMARK_ANDROGEN_RESPONSE	95	35	3.67	1.63E-08	1.17E-07
HALLMARK_UNFOLDED_PROTEIN_RESPONSE	108	36	3.15	1.96E-07	1.23E-06
HALLMARK_DNA_REPAIR	147	40	2.35	1.38E-05	7.68E-05
HALLMARK_ESTROGEN_RESPONSE_LATE	191	48	2.11	2.09E-05	0.00010425
HALLMARK_PROTEIN_SECRETION	94	26	2.40	0.000315891	0.001435869
HALLMARK_PI3K_AKT_MTOR_SIGNALING	98	26	2.26	0.000636708	0.002652949
HALLMARK_UV_RESPONSE_DN	138	33	1.97	0.000975424	0.00375163
HALLMARK_SPERMATOGENESIS	103	26	2.12	0.001411675	0.005041697

H1792 cells

Geneset	Size	Overlap	OddRatio	p-value	q-value
HALLMARK_MYC_TARGETS_V1	194	98	7.71	1.38E-39	6.92E-38
HALLMARK_G2M_CHECKPOINT	191	95	7.46	1.10E-37	2.75E-36
HALLMARK_E2F_TARGETS	195	90	6.45	1.49E-32	2.48E-31
HALLMARK_MTORC1_SIGNALING	196	85	5.75	2.05E-28	2.56E-27
HALLMARK_PROTEIN_SECRETION	95	41	5.64	2.31E-14	2.31E-13
HALLMARK_MITOTIC_SPINDLE	198	64	3.56	3.17E-14	2.64E-13
HALLMARK_ANDROGEN_RESPONSE	97	40	5.21	2.94E-13	2.10E-12

HALLMARK_UV_RESPONSE_DN	140	47	3.75	1.76E-11	1.10E-10
HALLMARK_OXIDATIVE_PHOSPHORYLATION	184	55	3.17	6.13E-11	3.40E-10
HALLMARK_UNFOLDED_PROTEIN_RESPONSE	109	39	4.13	1.03E-10	5.17E-10
HALLMARK_GLYCOLYSIS	199	57	2.99	1.76E-10	7.99E-10
HALLMARK_EPITHELIAL_MESENCHYMAL_TRANSITION	197	56	2.95	3.47E-10	1.45E-09
HALLMARK_ADIPOGENESIS	194	52	2.72	1.33E-08	5.10E-08
HALLMARK_HYPOXIA	194	49	2.51	2.52E-07	9.00E-07
HALLMARK_MYC_TARGETS_V2	58	22	4.52	3.67E-07	1.22E-06
HALLMARK_PI3K_AKT_MTOR_SIGNALING	103	30	3.04	2.33E-06	7.29E-06
HALLMARK_ESTROGEN_RESPONSE_EARLY	196	44	2.14	2.61E-05	7.67E-05
HALLMARK_TGF_BETA_SIGNALING	54	18	3.69	3.30E-05	9.16E-05
HALLMARK_NOTCH_SIGNALING	32	13	5.04	3.86E-05	0.000101647
HALLMARK_HEME_METABOLISM	194	41	1.98	0.000196865	0.000492161
HALLMARK_DNA_REPAIR	148	33	2.12	0.000286098	0.000681187
HALLMARK_FATTY_ACID_METABOLISM	157	34	2.04	0.00041426	0.000941501
HALLMARK_CHOLESTEROL_HOMEOSTASIS	73	19	2.60	0.000761123	0.001654616
HALLMARK_ESTROGEN_RESPONSE_LATE	197	39	1.82	0.001061313	0.002211068
HALLMARK_PEROXISOME	104	23	2.09	0.002491419	0.004982838
HALLMARK_WNT_BETA_CATENIN_SIGNALING	42	12	2.95	0.002978549	0.00572798
HALLMARK_TNFA_SIGNALING_VIA_NFKB	199	37	1.69	0.004295077	0.007953846
HALLMARK_APOPTOSIS	160	31	1.77	0.004553919	0.008131998

STable 9. Plasmid DNAs used for CRISPR cloning

Plasmid	Source	Identifier
psPAX2	Addgene	12260
pMD2.G	Addgene	12259
lentiCas9-Blast	Addgene	52962
hUBCp_Cas9_3xNLS_p2a_puroR	Addgene	81251
pLKO.1 -GFP	Brown Laboratory, ISMMS	N/A
pLKO.1 -mCherry	Addgene	128073

STable 10. Plasmid DNAs used for CRISPR cloning

	sgRNA Target Sequence	PAM sequence
AURKA_sgRNA#1	CCATATAGAAAATAATCCTG	AGG
AURKA_sgRNA#2	CCTGAAAACTCACCGAAGGT	GGG
AURKB_sgRNA#1	CATCAACCCATACTGCAGGT	GGG
AURKB_sgRNA#2	TCTTTCCGGAGGACTCGCTG	GGG
Non-target_sgRNA#1	AAAAAGCTTCCGCCTGATGG	N/A

Supplementary Notes

Supplementary Note 1. Unsupervised clustering of the 53 early-stage lung adenocarcinoma into Invasive and Indolent tumors

First, we performed an unsupervised hierarchical clustering for the 53 histologically heterogeneous LUAD using 1000 most variable genes (SFig. 1a, left). The unsupervised hierarchical clustering resulted in two distinct groups containing 20 and 33 samples, respectively. Samples in each group were mostly consistent with their histological subtypes; Group 1 on the left (red color in Inv. Class) was mostly aggressive tumors AC, MP, PAP and SOL while Group 2 on the right side (blue color in non-Inv. Class) was generally known to be non-invasive tumors such as MIA, AIS, and LPA. Therefore, we annotated Group 1 as "Invasive" and Group 2 as "Indolent". Then, differentially expressed genes (DEGs) between the Invasive and Indolent groups were determined based on t-test and a signature of 1,214 genes was obtained based on cutoffs (fold-change (FC) > 1.5 and FDR <0.01, SFig. 1a, right). When the samples were re-clustered using the 1,214 DEGs, one sample was switched from Indolent to Invasive group. DEGs were refined based on updated groups (21 Invasive vs. 32 Indolent tumors) and the 1,322 DEGs were finally identified, (Fig. 1a, Supplementary Data 1). Further re-clustering samples based on the updated DEGs yielded no additional group member change.

Supplementary Note 2. The signature genes independent from sex and smoking status

Because our dataset included a higher proportion of female patients (63%, Supplementary Table 1) that reflects current lung cancer epidemiology, we tested whether the signature genes were influenced by sex difference. Only 1 out of 1,322 genes was significantly associated with sex (FDR<0.01, Supplementary Note Fig. 1a). Cigarette smoking is the major cause of lung adenocarcinoma[1]. Since smoking status of the 53 patients was not available, we used RNAseq profiles of the CPTAC LUAD dataset to test whether the genes were associated with smoking status[2]. Sixty-one samples with stage I and II were separated into 22 more invasive and 39 less

invasive tumors based on unsupervised clustering using our signature genes and smoking status of the patients in each group was similar (Chi-square test p=0.77, Supplementary Note Fig. 1b) suggesting that the smoking status is not associated with the invasiveness signature genes. This result is consistent with a recent report that the smoking signature activity did not differ between AIS/MIA and invasive LUADs[3]. Taken together, gene expression variances of the invasiveness signature genes were not significantly associated with sex nor smoking status of the patients.

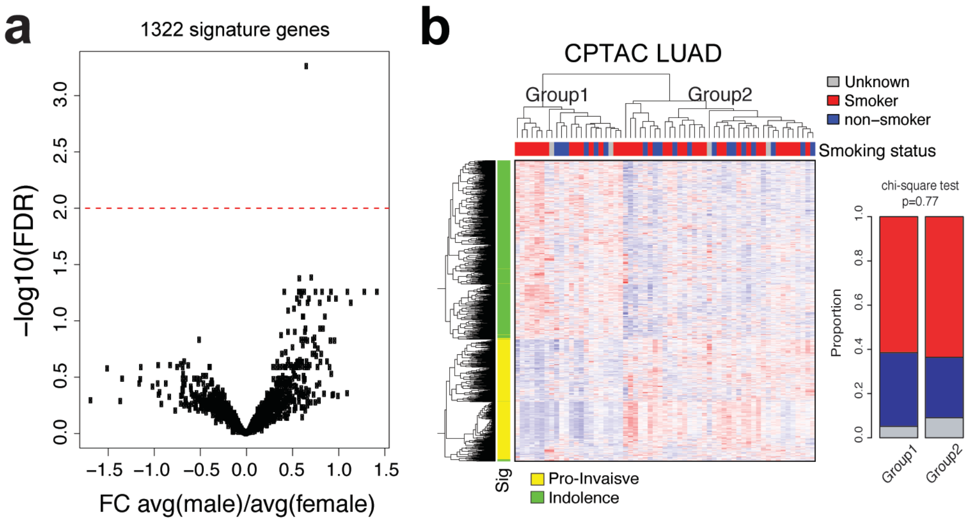

Supplementary Note Fig. 1. Expression of the signature genes independent from sex or smoking status of patients. A. The association of expression of 1322 genes with sex of 53 patients. Two-side t-test p-values between male and female were adjusted (FDR) and only one gene was significant (FDR<0.01). **b.** CPTAC LUAD samples (stage I and II tumors) clustered by the signature genes. Proportions of smokers were similar in both groups (chi-square test p-value=0.77).

Supplementary Note 3. Comparison of functional enrichments based on the invasive signature or histology-based DEGs

Unsupervised clustering based on gene expression profiles provided a greater number of differentially expressed genes between invasive and indolent tumors than histology-based clustering. To test whether the increased genes are biologically meaningful, we evaluated FET p-

values against gene sets in Fig. 1d using the histology based DEGs (SFig. 1c) and compared them with the results using our signature genes. Because most of histology based DEGs were included in the signature genes (Fig. 1c), it was somewhat expected to observe similar enrichment patterns from the approaches (SFig. 1f). Several gene sets were similarly enriched for genes by both approaches including cell cycle related gene sets (FET p= 2.9×10^{-52} and 2.2×10^{-51} for G2M_CHECKPOINT, 1.1×10^{-52} and 7.8×10^{-50} for E2F_TARGETS from our signature genes and histology-based DEGs, respectively). However, the pro-invasive and indolence signatures showed more significant associations with gene sets specific for EMT (FET p=9.9×10^{-29} and 5.810^{-14} from pro-invasive and histology-based DEGs, respectively, SFig. 1f), Invasion (FET p=1.5×10^{-25} and 2.6×10^{-13}, respectively), or tumor suppressor genes (FET p=3.5×10^{-8} and 7.1×10^{-5} from indolent and histology-based DEGs, respectively, SFig. 1f). This indicates that the biological information revealed by the invasiveness signature supplements the mechanistic and prognostic information provided histology alone DEGs. Moreover, when we exclusively included genes in the invasiveness signature DEGs but not in histology-based DEGs, significant enrichments were still observed for some of key pathways such as EMT or tumor suppressor genes (SFig. 1g). These results support our rationale to use the invasiveness signature genes from gene expression based clustering to biologically and functionally classify early lung adenocarcinoma specimens.

Supplementary Methods

Quality control of RNAseq data

Raw data (in fastq format) of 53 samples were processed through Tophat and Cufflink[4] using hg19 reference genome and UCSC refseq gtf. The average number of reads per samples is 45 million reads with the minimum around 28 million (Supplementary Method Fig. 1a). The FPKM of 18,457 genes estimated by Cufflink showed median expression of 3 (log2(FPKM)) (Supplementary Method Fig. 1b). Using the processed FPKM value, we confirmed that sex of each patient was consistent with predicted sex based on expression of *RPS4Y1*, a sex-specific gene on Y chromosome[5] (Supplementary Method Fig. 1c).

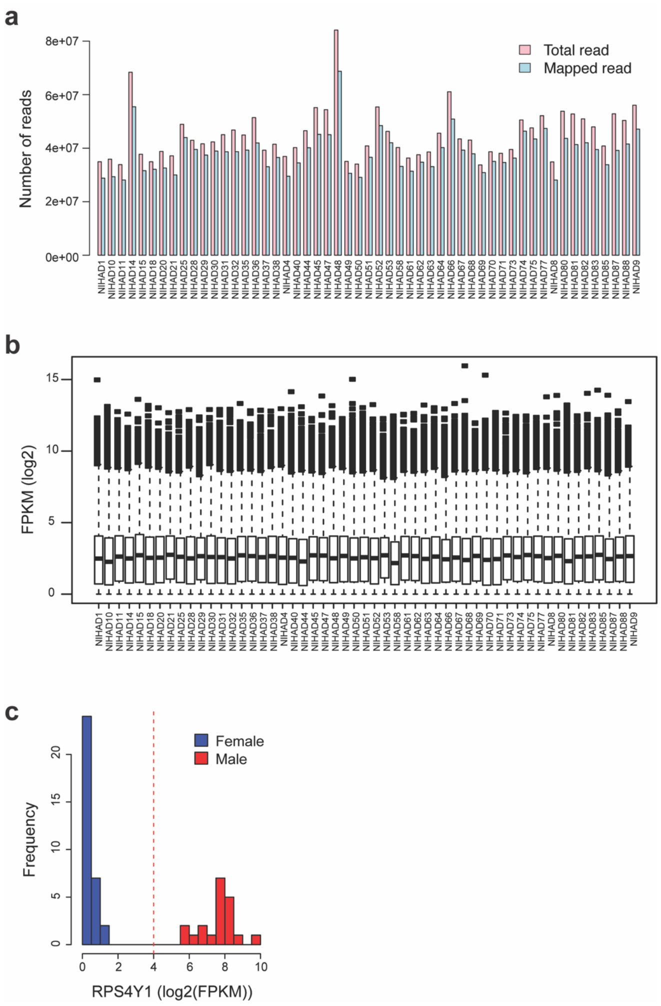

Supplementary Method Fig. 1. Quality check of RNAseq data of 53 esLUAD patients. a. A barplot of number of reads; total number of reads (red) and uniquely mapped reads to the reference genome (blue). **b.** A boxplot showing gene expression distribution of 18457 genes in each sample. Band indicates median value of log2(FPKM) and the box edges are the 25th and 75th percentiles for each sample. Whiskers indicate maximum and minimum values of each group except for outliers. **c.** Expression distributions of sex-specific gene *RPS4Y1* based on sex reported in clinical data.

Patient-centric multi-Omics data QC for CNV, DNA methylation and RNAseq data from TCGA LUAD stage I data

While integration of different molecular data enhances our understanding of molecular mechanisms underlying complex biological systems, large scale omics data sometimes contain sample labeling errors[5]. Therefore, we performed sample alignment to filter out any potential mis-labeled samples among CNV, methylation and RNAseq data from TCGA before we used them for network construction. We collected 218 samples with CNV, methylation, and RNAseq profiles available and performed pairwise alignment between RNAseq-CNV and RNAseq-methylation using MODMatcher[5]. For each pairwise alignment, we first measured the correlation of cis genes between two molecular data and identified top 1000 most significant gene; positively associated genes in RNAseq-CNV and negatively associated genes in RNAseq-methylation. Then, values of the selected cis genes were rank-transformed, and sample-wise correlation were measured as sample similarity score. If a sample is well aligned between two molecular data, the sample similarity score is expected to be higher than random pairing as shown in an example (Supplementary Method Fig. 2a). We confirmed samples between RNAseq and CNV had perfectly aligned each other. From the RNAseq-methylation alignment, sample similarity scores of two samples (TCGA-49-4514-01A and TCGA-97-8177-01A) were not clearly separated from null distribution (Supplementary Method Fig. 2b-c) so we removed these in further analysis.

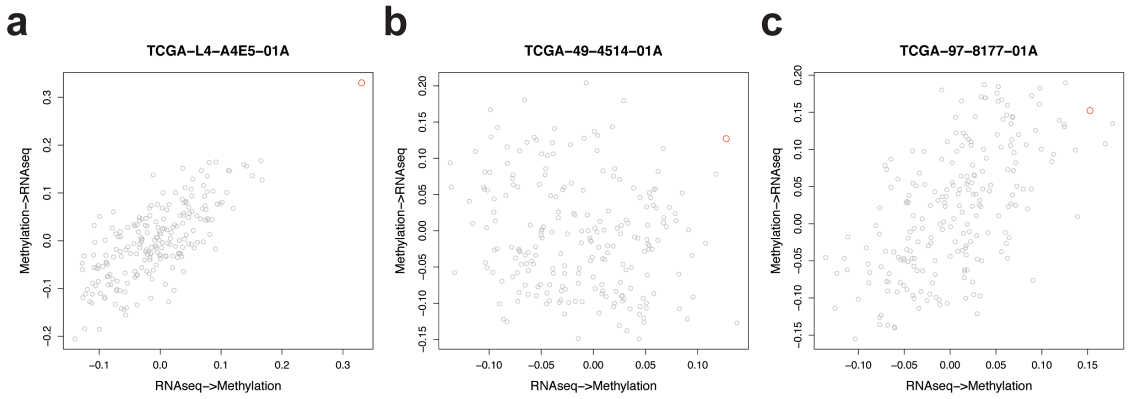

Supplementary Method Fig. 2. Sample similarity scores from RNAseq-methylation alignment. a. An example of well-aligned case. The red dot (self-similarity score) is distinctly separated from sample score with other samples. **b.** Ambiguous sample (TCGA-49-4514-01A) with poor self-similarity score compared with other samples. **c.** Ambiguous sample (TCGA-97-8177-01A) with poor self-similarity score.

Preparation for network construction

The 216 samples confirmed by sample alignment were further used to construct a Bayesian network of esLUAD[6, 7]. First, we selected genes with higher expression (log2(RSEM)>4.5, Supplementary Method Fig. 3a) and larger variances (variance > 0.6, Supplementary Method Fig. 3b). A total of 8,533 informative genes with detectable expression levels and large variances across samples were selected to be included in the network reconstruction process (Supplementary Method Fig. 3c). Among them, the expression of 3,476 and 761 genes was cis-regulated by CNVs or promoter methylation (FDR < 0.01), respectively, and cis-CNVs and cis-methylations were included as root nodes in the network construction.

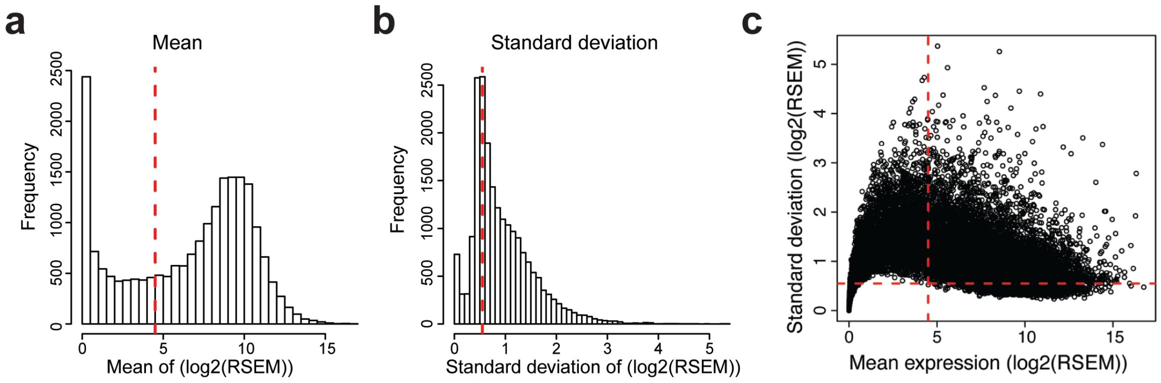

Supplementary Method Fig. 3. Selection of genes for network reconstruction. a. Higher expressed genes were first selected (mean expression > 4.5). **b.** Genes with little variances were filtered out due to less information (variance > 0.6). **c.** Scatter plot of mean expression and variance of all genes.

Western blot

For synchronization, cells were treated with 400ng/ml nocodazole for 16-20hr and were harvested to obtain total protein extract for Western blot. Whole cell protein extract was prepared from cells using RIPA lysis buffer (Thermo Scientific). Protein concentration was estimated using Pierce BCA protein assay kit (Thermo Scientific) and 40µg of protein was boiled in Laemmli's SDS sample buffer (Boston bioproducts) to run on SDS-PAGE gel. Protein was electro-transferred to PVDF membrane, blocked with 5% non-fat powdered milk (Boston bioproducts), followed by overnight incubation with primary antibody at 4°C. The membrane was washed thrice with 0.05% Tris-buffered saline Tween-20 (TBST) wash buffer for 10 min each and incubated with HRP-conjugated secondary antibody. Membrane was then washed three times with TBST and developed with Clarity Western ECL substrate (Bio-Rad Laboratories). The activities of AURKA and AURKB, measured as expression

of p-Aur-A (T288) and pAur-B (T232) was significantly suppressed in all 3 cell lines with both drugs (Supplementary Method Fig. 4a-c).

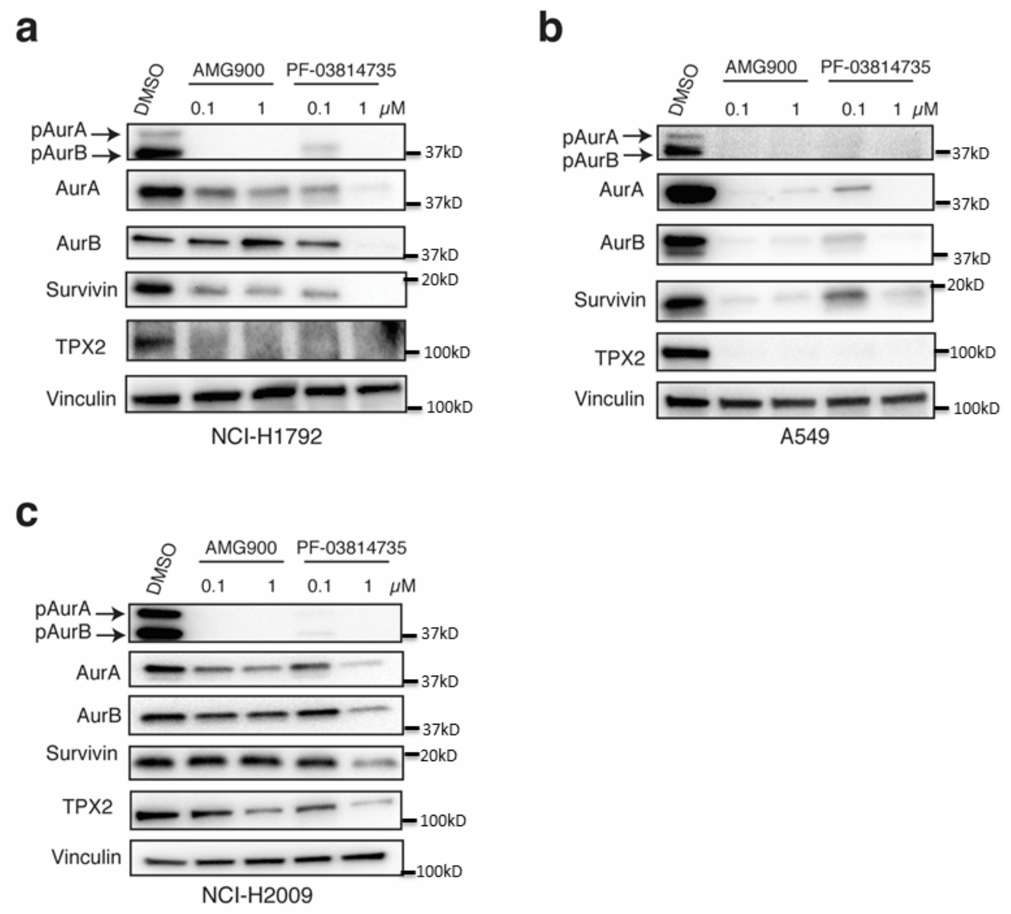

Supplementary Method Fig. 4. Pan aurora kinase inhibitors suppress aurora kinases activity of invasive LUAD cells a-c. Western blot for indicated proteins expression in **a.** H1792, **b.** A549 and **c.** H2009 on treatment with indicated concentrations of AMG900 and PF-03814735 at 48hr.

CD31, E-Cadherin and Vimentin Immunohistochemistry and Masson's Trichrome staining

Immunohistochemistry for CD31 in mouse lung sections was performed on Leica Bond III with antigen retrieval for 10 minutes using citrate buffer (pH6). Slides were then incubated with primary antibody for 30 minutes at room temperature and detected using an HRP conjugated compact polymer system with DAB as the chromogen and was counterstained with hematoxylin.

Immunohistochemistry for E-cadherin (retrieval Tris-EDTA buffer pH9) was performed on 5 micron sections using a Leica Bond II autostainer. Twelve regions of interest were imaged per condition (vehicle and AMG900 treatment) and analyzed using the IHC Profiler and plugin for Image J.

For immuno-histochemical staining for pAKT, sections were deparaffinized, rehydrated with xylene and descending grades of alcohol and water. For antigen retrieval slides were boiled in Antigen Unmasking Solution (Vector Labs, CA) for 30 min in a steamer. After cooling, slides were incubated for 5 min in 3% H2O2 (in ethanol) to quench endogenous peroxidase activity. Blocking was performed using the Vectastain ABC Elite Kit (Vector labs, CA). Sections were incubated with the primary antibodies overnight at 4°C. Sections were washed and incubated with appropriate secondary antibodies from the ABC kit and staining was revealed using the ImmPACT DAB kit (Vector Labs, CA). Slides were counterstained in hematoxylin, dehydrated and mounted. For collagen quantitation, lung sections were stained with trichrome stain as described previously[8]. For each tumor, trichrome positive area was marked using Aperio ImageScope 12.1 software. Collagen positive area was normalized to tumor area for each section and plotted.

Viability assay

For IC_{50} 1-2 X 10^3 cells were seeded in triplicate wells in a 96-well plate for treatment with serial dilutions of AMG900 (Apexbio) and PF-03814735 (Apexbio). Effect of both drugs on the viability of lung adenocarcinoma cells was tested at 48h after drug treatment using alamarBlue™ Cell Viability Reagent. IC_{50} value for the effect of drugs on cell viability was calculated by plotting log inhibitor *vs* normalized response- variable slope. Additionally, all cells treated with aurora kinase inhibitors demonstrated suppressed proliferation rate over the course of longer time duration (Supplementary Method Fig. 5).

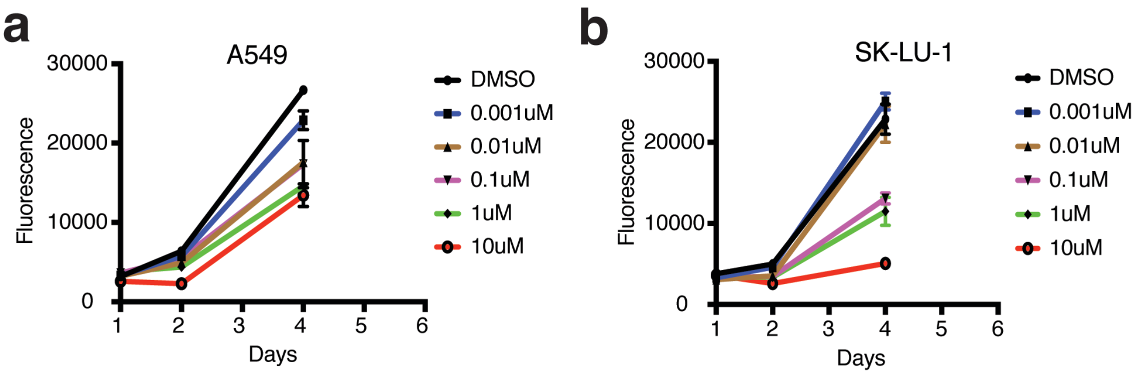

Supplementary Method Fig. 5. Cell growth rate is suppressed over long term with aurora kinase inhibitors. Cell growth assay shown as relative fluorescence of A549 (a) and SK-LU-1 cells (b) treated with DMSO vs serial dilution of AMG900 or PF-03814735 measured through alamar blue assay at Day1, 2, and 4. n=3, Data presented as mean ±s.e.m.

List of antibodies used for western blot analysis and IHC is in Supplementary Method Table 1.

Supplementary Method Table 1. List of antibodies

Antibody	Source	Dilution used	Identifier

Aurora A (WB)	Cell Signaling Technology	1:1000	14475
Aurora A (IHC)	Abcam	1:400	ab13824
Aurora B/AIM1 (WB)	Cell Signaling Technology	1:1000	3094
Aurora B (IHC)	Abcam	1:100	ab2254
CD31 (IHC)	Abcam	1:100	ab182981
p-Aurora A/B/C	Cell Signaling Technology	1:2000	2914
E-Cadherin (IHC)	Abcam	1:1000	ab53033
TPX2	Cell Signaling Technology	1:1000	8559
TPX2	Novus	1:1000	NB500-179
Survivin	Cell Signaling Technology	1:1000	2808
p-Akt	Cell Signaling Technology	1:1000	13038
Akt	Cell Signaling Technology	1:1000	9272
pAKT (IHC)	Cell Signaling Technology		4060
p-ERK1/2	Cell Signaling Technology	1:1000	9101
ERK1/2	Cell Signaling Technology	1:1000	4695
p-mTOR	Cell Signaling Technology	1:1000	5536
mTOR	Cell Signaling Technology	1:1000	2983
p-p70 S6 Kinase (Ser371)	Cell Signaling Technology	1:1000	9208
p-p70 S6 Kinase (Thr389)	Cell Signaling Technology	1:1000	9234
p-4E-BP1	Cell Signaling Technology	1:1000	2855
E-Cadherin	Cell Signaling Technology	1:1000	3195
N-Cadherin	Cell Signaling Technology	1:1000	13116
Vimentin	Cell Signaling Technology	1:1000	5741
Claudin-1	Cell Signaling Technology	1:1000	13255
Slug	Cell Signaling Technology	1:1000	9585
Snail	Cell Signaling Technology	1:1000	3879
β-Actin	Sigma	1:5000	A5316
Vinculin	Sigma	1:20,000	V4505

Uncropped blots for Supplementary Figures

SFig. 7a Western blot for H1792 cells transduced with indicated sgRNAs

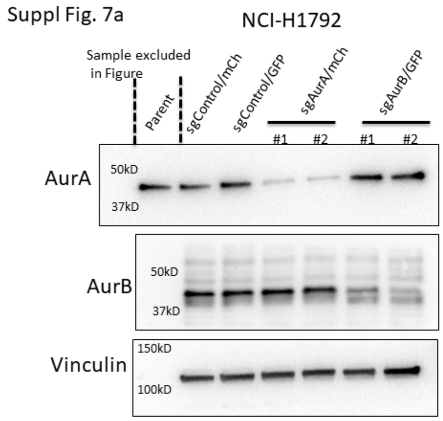

SFig. 8a Western blot for H1792 cells transduced with indicated sgRNAs

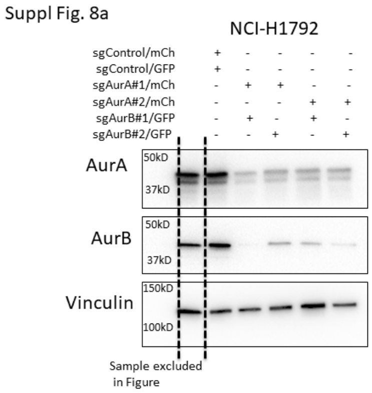

SFig. 8e Western blot for H1792 cells transduced with indicated sgRNAs at day 2 and day8

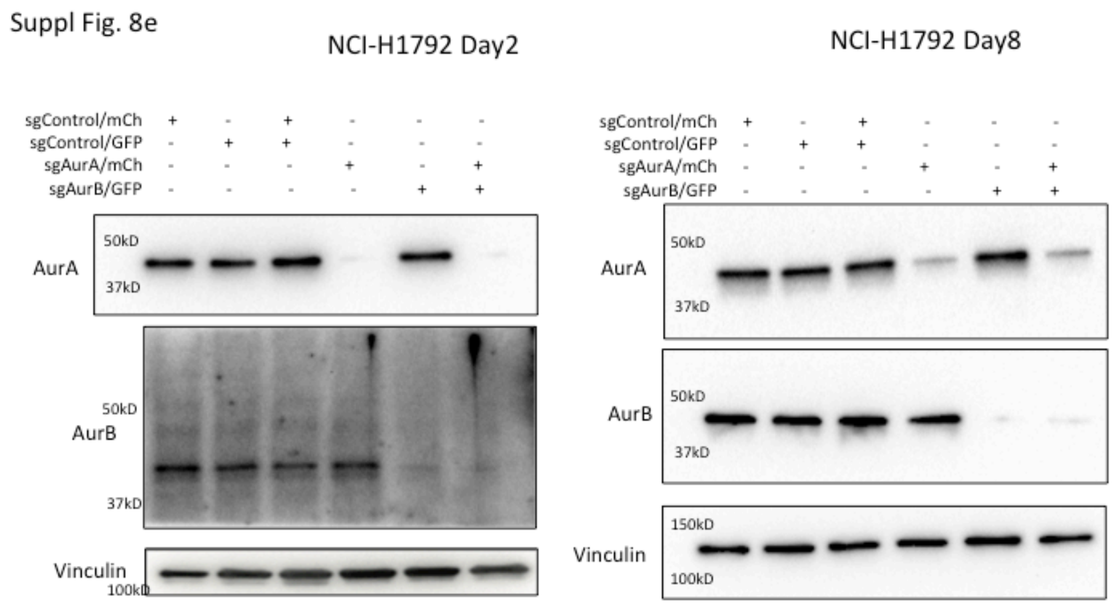

SFig. 10d Western blot for indicated proteins in H1792 cells treated with DMSO and indicated concentrations of AMG900 for 48hr.

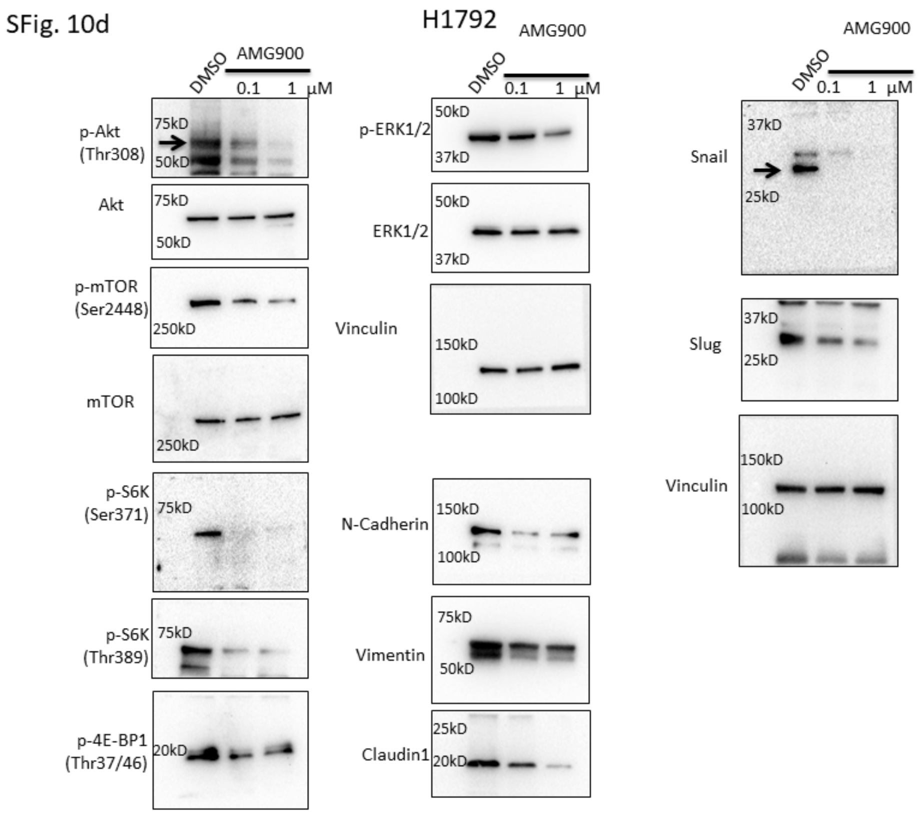

SFig. 10e Western blot for H1792 cells treated with indicated sgRNAs for AKT/mTOR and EMT pathway.

SFig. 10e

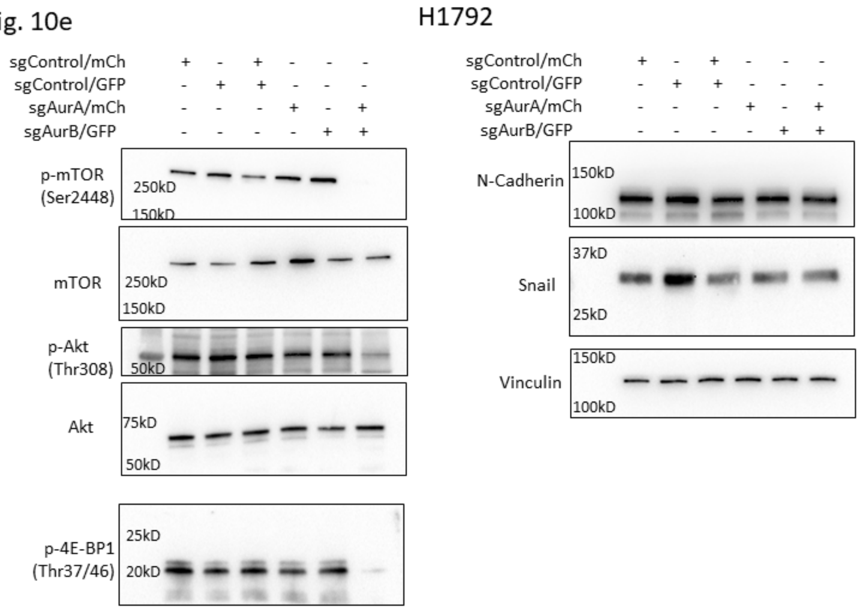
H1792
sgControl/mCh + - + - - -
sgControl/GFP - + + - - -
sgAurA/mCh - - - + - +
sgAurB/GFP - - - - + +
p-mTOR (Ser2448)
250kD
150kD
mTOR
250kD
150kD
p-Akt (Thr308)
50kD
Akt
75kD
50kD
p-4E-BP1 (Thr37/46)
25kD
20kD
N-Cadherin
150kD
100kD
Snail
37kD
25kD
Vinculin
150kD
100kD

Supplementary References

1. Cancer Genome Atlas Research N. Comprehensive molecular profiling of lung adenocarcinoma. Nature 2014;511:543-50.
2. Gillette MA, Satpathy S, Cao S, et al. Proteogenomic Characterization Reveals Therapeutic Vulnerabilities in Lung Adenocarcinoma. Cell 2020;182:200-225 e35.
3. Chen H, Carrot-Zhang J, Zhao Y, et al. Genomic and immune profiling of pre-invasive lung adenocarcinoma. Nat Commun 2019;10:5472.
4. Trapnell C, Roberts A, Goff L, et al. Differential gene and transcript expression analysis of RNA-seq experiments with TopHat and Cufflinks. Nat Protoc 2012;7:562-78.
5. Yoo S, Huang T, Campbell JD, et al. MODMatcher: multi-omics data matcher for integrative genomic analysis. PLoS Comput Biol 2014;10:e1003790.
6. Zhu J, Zhang B, Smith EN, et al. Integrating large-scale functional genomic data to dissect the complexity of yeast regulatory networks. Nat Genet 2008;40:854-61.
7. Liu Y, Yu H, Yoo S, et al. A Network Analysis of Multiple Myeloma Related Gene Signatures. Cancers (Basel) 2019;11.
8. Borczuk AC, Sole M, Lu P, et al. Progression of human bronchioloalveolar carcinoma to invasive adenocarcinoma is modeled in a transgenic mouse model of K-ras-induced lung cancer by loss of the TGF-beta type II receptor. Cancer Res 2011;71:6665-75.
