# Supplementary material for: Integrative network analysis of early-stage lung adenocarcinoma identifies aurora kinase inhibition as interceptor of invasion and progression
Source: Nat Commun. 2022 Mar 24;13:1592. doi: 10.1038/s41467-022-29230-7 (PMC8948234; doi:10.1038/s41467-022-29230-7)
Supplement: Supplementary file 3 — Description to Supplementary Information [file 41467_2022_29230_MOESM3_ESM.pdf]

Supplementary Data Legends

Supplementary Data 1. List of 1322 DEGs between invasive and non-invasive tumors

Supplementary Data 2. Enrichment test against GO terms using pro-invasive and indolent signatures

Supplementary Data 3. Enrichment test against Hallmark and selected C2 gene sets using invasive and non-invasive signatures

Supplementary Data 4. Differentially expressed genes determined by DEseq2 (FDR<0.01)
