# Supplementary material for: Integrative network analysis of early-stage lung adenocarcinoma identifies aurora kinase inhibition as interceptor of invasion and progression
Source: Nat Commun. 2022 Mar 24;13:1592. doi: 10.1038/s41467-022-29230-7 (PMC8948234; doi:10.1038/s41467-022-29230-7)
Supplement: Supplementary file 8 — Reporting Summary [file 41467_2022_29230_MOESM8_ESM.pdf]

nature research

Corresponding author(s): Jun Zhu, Charles A. Powell

Last updated by author(s): Feb 12, 2022

Reporting Summary

Nature Research wishes to improve the reproducibility of the work that we publish. This form provides structure for consistency and transparency in reporting. For further information on Nature Research policies, see our Editorial Policies and the Editorial Policy Checklist.

Statistics

For all statistical analyses, confirm that the following items are present in the figure legend, table legend, main text, or Methods section.

n/a	Confirmed	
☐	☒	The exact sample size (*n*) for each experimental group/condition, given as a discrete number and unit of measurement
☐	☒	A statement on whether measurements were taken from distinct samples or whether the same sample was measured repeatedly
☐	☒	The statistical test(s) used AND whether they are one- or two-sided<br>*Only common tests should be described solely by name; describe more complex techniques in the Methods section.*
☐	☒	A description of all covariates tested
☐	☒	A description of any assumptions or corrections, such as tests of normality and adjustment for multiple comparisons
☐	☒	A full description of the statistical parameters including central tendency (e.g. means) or other basic estimates (e.g. regression coefficient) AND variation (e.g. standard deviation) or associated estimates of uncertainty (e.g. confidence intervals)
☐	☒	For null hypothesis testing, the test statistic (e.g. *F*, *t*, *r*) with confidence intervals, effect sizes, degrees of freedom and *P* value noted<br>*Give P values as exact values whenever suitable.*
☐	☒	For Bayesian analysis, information on the choice of priors and Markov chain Monte Carlo settings
☐	☒	For hierarchical and complex designs, identification of the appropriate level for tests and full reporting of outcomes
☐	☒	Estimates of effect sizes (e.g. Cohen's *d*, Pearson's *r*), indicating how they were calculated

Our web collection on statistics for biologists contains articles on many of the points above.

Software and code

Policy information about availability of computer code

Data collection	Publicly available gene expression profiles with GEO accession numbers were downloaded using a "getGEO" command in a R package "GEOquery (Version 2.62.0)". CNV, DNA methylation, and RNAseq of TCGA LUAD were downloaded using "gdc-client" after creating a manifest file for the samples of interest. For other data, no specific codes or programs were necessary for collection.
Data analysis	R (version 4.0.1) was used for most of data analysis. DESeq2 (R package version 1.34.0) was used to identify differentially expressed genes between DMSO and AMG900 treated cells. Caret (R package version 6.0-90) and Glmnet (R version (4.1.1) were used in elastic network modeling for IVS based on invaisveness signature genes. Integrative regulatory network was viewed and arranged using CytoScape (version 3.8.1). All codes and scripts used in this study are available in Dr. Zhu's lab git repository https://github.com/integrativenetworkbiology/Tumor_invasion_esLUAD. For image data from migration, invasion, and wound healing assay with aurora kinase inhibition, ImageJ (version 1.52) was used.

For manuscripts utilizing custom algorithms or software that are central to the research but not yet described in published literature, software must be made available to editors and reviewers. We strongly encourage code deposition in a community repository (e.g. GitHub). See the Nature Research guidelines for submitting code & software for further information.

Data

Policy information about availability of data

All manuscripts must include a data availability statement. This statement should provide the following information, where applicable:

- Accession codes, unique identifiers, or web links for publicly available datasets
- A list of figures that have associated raw data
- A description of any restrictions on data availability

RNA sequencing data generated in this study is deposited in GEO database with the accession code GSE166722. Other publicly available dataset used in this study is

GSE27717 (Kras/Tgfbr2 mouse model), GSE68465 (Shedden et al.), GSE31210 (Okayama et al.), GSE50081 (Der et al.), GSE42127 (Tang et al.), GSE30219 (Rousseaux et al.), and GSE26939 (Wilkerson et al.). RNAseq, CNV, and DNA methylation profiles of TCGA Stage I and II LUAD samples were downloaded by GDC data portal (https://portal.gdc.cancer.gov/projects/TCGA-LUAD/). RNAseq of 70 LUAD cancer cells were downloaded by CCLE Data section (https://depmap.org/portal/download/?release=CCLE+2019&release=Fusion&release=DNA+Copy_Number). Reference genome and transcriptome, hg19 reference genome and UCSC refseq gtf files were downloaded (hgdownload.soe.ucsc.edu/goldenPath/hg19/bigZips). No restriction of data used in this study.

Field-specific reporting

Please select the one below that is the best fit for your research. If you are not sure, read the appropriate sections before making your selection.

☒ Life sciences ☐ Behavioural & social sciences ☐ Ecological, evolutionary & environmental sciences

For a reference copy of the document with all sections, see nature.com/documents/nr-reporting-summary-flat.pdf

Life sciences study design

All studies must disclose on these points even when the disclosure is negative.

Sample size

No specific sample size calculation was performed. Tumor speciments were acquired from 53 patients of resected lu ng adenocarcinoma with hisgoloty classfication that included adenocarcinoma in situ and minimally invasive adenocarcinoma. To validate invasiveness signature in in-vitro model, 8 cell lines (5 Kras mutant and 3 Kras wildtype) from more invasive and 5 from less invasive cell lines were selected. CRISPR knockout assays were tested in 2 representative cell lines (H1792 and A549) each with 2 sgRNAs for AURKA and AURKB. Drug phenotype assays were validated in 5 more invasive cell lines with 2 aurora kinase inhibitors. For cell lines RNAseq, we used 4 and 4 replicates for A539 and H1792 cell lines. Singliang validation by western blot was done in the two cell lines (A549 and H1792). For in-vivo Adeno cre mouse model, we used 10 vehicles and 8 AMG900 treated animals. Micro CT data was quantified in n=3 per treatment group. For histopathological analysis, IHC staining and Masson's trichome starning (n=2) was used per treatment group. For all phenotype assay, mulitple samples (n>3) were used. Sample size were chosen to support necessary requirements for determining statitical significance and to generalize observations without being biased to a single condition such as cell line, driver mutation, or a specific compound.

Data exclusions

No sample generated in this study was excluded from the analysis. For publicly available LUAD dataset, we focused on Stage I and II tumors to be consistent with our interest in early-stage LUAD. The Bayesian network was construct using Stage I tumors only to focus on gene regulations at early stage.

Replication

All phenotype assays were performed in 3 independent trials. All attempts were successful. RNAseq for cell lines and animal experiments were replicated 2 times and all attempts were successful.

Randomization

Allocation of animals to treatment was random. In all other experiments, samples were allocated randomly.

Blinding

Histological assessment was performed blindly by a pathologist then histopathological subtypes of the 53 resected tumors were collected. But that information was not used to classify the 53 patients into invasive and non-invasive tumors based on unsupervised clustering. Hence the molecular based clustering and histopathological subtypes are independent. The other in-vitro results were not performed blindly as they were performed by individual researchers and do not compass subjective measurements.

Reporting for specific materials, systems and methods

We require information from authors about some types of materials, experimental systems and methods used in many studies. Here, indicate whether each material, system or method listed is relevant to your study. If you are not sure if a list item applies to your research, read the appropriate section before selecting a response.

Materials & experimental systems

n/a	Involved in the study	
☐	☒	Antibodies
☐	☒	Eukaryotic cell lines
☒	☐	Palaeontology and archaeology
☐	☒	Animals and other organisms
☐	☒	Human research participants
☒	☐	Clinical data
☒	☐	Dual use research of concern

Methods

n/a	Involved in the study	
☒	☐	ChIP-seq
☒	☐	Flow cytometry
☒	☐	MRI-based neuroimaging

Antibodies

Antibodies used

Aurora A (WB; Cell Signaling Technology; 14475; 1:1000), Aurora A (IHC; Abcam; ab13824; 1:400), Aurora B/AIM1 (WB; Cell Signaling Technology; 3094; 1:1000), Aurora B (IHC; Abcam; ab2254; 1:100), CD31 (Abcam; ab182981; 1:100), p-Aurora A/B/C (Cell Signaling Technology; 2914; 1:2000), E-Cadherin (IHC; Abcam; ab53033; 1:1000), TPX2 (Cell Signaling Technology; 8559; 1:1000), TPX2 (Novus; NB500-179; 1:1000), Survivin (Cell Signaling Technology; 2808; 1:1000), p-Akt (Cell Signaling Technology; 13038; 1:1000), Akt (Cell Signaling Technology; 9272; 1:1000), pAKT

(IHC; Cell Signaling Technology; 4060; 1:100), p-ERK1/2 (Cell Signaling Technology; 9101; 1:1000), ERK1/2 (Cell Signaling Technology; 4695; 1:1000), p-mTOR (Cell Signaling Technology; 5536; 1:1000), mTOR (Cell Signaling Technology; 2983; 1:1000), p-p70 S6 Kinase (Ser371) (Cell Signaling Technology; 9208; 1:1000), pp70 S6 Kinase (Thr389) (Cell Signaling Technology; 9234; 1:1000), p-4E-BP1 (Cell Signaling Technology; 2855; 1:1000), E-Cadherin (WB; Cell Signaling Technology; 3195; 1:1000), N-Cadherin (Cell Signaling Technology; 13116; 1:1000), Vimentin (Cell Signaling Technology; 5741; 1:1000), Claudin-1 (Cell Signaling Technology; 13255; 1:1000), Slug (Cell Signaling Technology; 3879; 1:1000), β-
Actin (Sigma; A5316; 1:5000), Vinculin
9585; 1:1000), Snail (Cell Signaling
Technology; (Sigma; V4505; 1:20,000),
Antibody dilution for western blots were as specified by manufacturer. Antibody dilutions for IHC were used as per standardized and validated by pathology core at Weill Cornell Medicine, NY.
Peroxidase AffiniPure Goat Anti-Rabbit IgG (H+L) (Jackson lab; 111-035-144; 1:5000), AffiniPure Goat Anti-Mouse IgG (H+L) (Jackson lab; 115-005-062; 1:5000). List of antibodies is detailed in Supplementary Method Table1.

Validation

Antibody dilution for western blots were as specified by manufacturer. Antibody dilutions for IHC were used as per standardized and validated by pathology core at Weill Cornell Medicine, NY.

Eukaryotic cell lines

Policy information about cell lines

Cell line source(s)

Lung adenocarcinoma cell lines H1373, H1792, H2009, H1755, H1975, H1650, HCC-78, H3255, Calu-3, HCC-1833, HCC2279, SK-LU-1, A549 and HEK293T were used in the study. HCC-78 was obtained from DSMZ - German Collection of Microorganisms and Cell Cultures GmbH, Braunschweig, Germany. HCC-1833 was obtained from Korean cell line bank, Seoul, Korea. All remaining cell lines used in the study were obtained from American type culture collection (Manassas, VA)

Authentication

All cell lines were authenticated by short tandem repeat {STR) profiling from Genomics - Research Technology Support Facility, MICHIGAN STATE UNIVERSITY.

Mycoplasma contamination

All cell lines were periodically tested for mycoplasma contamination using mycoAlert Detection Kit (Lonza). None of the cell lines were contaminated.

Commonly misidentified lines
(See ICLAC register)

No commonly misidentified cell lines were used in this study.

Animals and other organisms

Policy information about studies involving animals; ARRIVE guidelines recommended for reporting animal research

Laboratory animals

All animals used in the study (LSL-KrasG12D positive, Tgfbr2flox/flox and Kras(G12D); TGFBR2-/- were C57 /B16 male/female of 6-8 weeks age.

Wild animals

No wild animals were used in this study.

Field-collected samples

Study did not involve sample collected from field.

Ethics oversight

All studies involving animals were performed under a protocol approved by the Icahn school of Medicine at Mount Sinai, Institutional Animal Care and Use Committee (IACUC LA11-00201).

Note that full information on the approval of the study protocol must also be provided in the manuscript.

Human research participants

Policy information about studies involving human research participants

Population characteristics

The demographic characteristics of the 53 patients are provided in Supplementary Table 1. Age or sex are not significantly associated invasivness of patients. EGFR mutation was enriched within the non-invasive patients while other key driver mutations (KRAS or TP53) were not different among both patient groups. There is non confounding factors such as current diagnosis or treatment after surgery.
The tissue microarray patients showed similar demographic characteristics such as age and sex as our RNAseq cohort. Genotype information of the cohort is not available.

Recruitment

Tumor tissues for RNAseq were collected from 2000-2010. All cases of AIS, MIA and LPA for which frozen tissue was collected were included without any selection.

Ethics oversight

The 53 frozen tumors tissues collected from 2000-2010 were retrieved for use of deidentified human tissue with clinical annotation as part of an Institutional Review Board (IRB) approved protocol of the Columbia University Cancer Center Tissue Bank (IRB #: AAAA-3987) that had waivers for consent.
All TMA materials were accessed through the Columbia University Cancer Center Tissue Bank IRB (IRB #: AAAA-3987) and the Weill Cornell Thoracic Surgery Biobank IRB (IRB #: 1008011221) approved retrospective human tissue protocols and were constructed under those protocols that had waivers for consent. These specimens were not acquired from clinical trials and there is no provision for reidentification after the TMAs were constructed.

Note that full information on the approval of the study protocol must also be provided in the manuscript.
